# Insights into acetate toxicity in *Zymomonas mobilis* 8b using different substrates

**DOI:** 10.1186/s13068-014-0140-8

**Published:** 2014-09-30

**Authors:** Shihui Yang, Mary Ann Franden, Steven D Brown, Yat-Chen Chou, Philip T Pienkos, Min Zhang

**Affiliations:** National Bioenergy Center, National Renewable Energy Laboratory, Golden, CO 80401 USA; Biosciences Division, Oak Ridge, TN 37831 USA; BioEnergy Science Center, Oak Ridge National Laboratory, Oak Ridge, TN 37831 USA

## Abstract

**Background:**

Lignocellulosic biomass is a promising renewable feedstock for biofuel production. Acetate is one of the major inhibitors liberated from hemicelluloses during hydrolysis. An understanding of the toxic effects of acetate on the fermentation microorganism and the efficient utilization of mixed sugars of glucose and xylose in the presence of hydrolysate inhibitors is crucial for economic biofuel production.

**Results:**

A new microarray was designed including both coding sequences and intergenic regions to investigate the acetate stress responses of *Zymomonas mobilis* 8b when using single carbon sources of glucose or xylose, or mixed sugars of both glucose and xylose. With the supplementation of exogenous acetate, 8b can utilize all the glucose with a similar ethanol yield, although the growth, final biomass, and ethanol production rate were reduced. However, xylose utilization was inhibited in both media containing xylose or a mixed sugar of glucose and xylose, although the performance of 8b was better in mixed sugar than xylose-only media. The presence of acetate caused genes related to biosynthesis, the flagellar system, and glycolysis to be downregulated, and genes related to stress responses and energy metabolism to be upregulated. Unexpectedly, xylose seems to pose more stress on 8b, recruiting more genes for xylose utilization, than does acetate. Several gene candidates based on transcriptome results were selected for genetic manipulation, and a TonB-dependent receptor knockout mutant was confirmed to have a slight advantage regarding acetate tolerance.

**Conclusions:**

Our results indicate *Z. mobilis* utilized a different mechanism for xylose utilization, with an even more severe impact on *Z. mobilis* than that caused by acetate treatment. Our study also suggests redox imbalance caused by stressful conditions may trigger a metabolic reaction leading to the accumulation of toxic intermediates such as xylitol, but *Z. mobilis* manages its carbon and energy metabolism through the control of individual reactions to mitigate the stressful conditions. We have thus provided extensive transcriptomic datasets and gained insights into the molecular responses of *Z. mobilis* to the inhibitor acetate when grown in different sugar sources, which will facilitate future metabolic modeling studies and strain improvement efforts for better xylose utilization and acetate tolerance.

**Electronic supplementary material:**

The online version of this article (doi:10.1186/s13068-014-0140-8) contains supplementary material, which is available to authorized users.

## Background

Lignocellulosic biomass is considered to be a renewable and sustainable resource to address global challenges on environmental protection, energy security, and economic development, and cellulosic ethanol production has made significant progress at the pilot and demonstration scales. However, the toxic compounds generated during the deconstruction processes of pretreatment and enzymatic saccharification to release fermentable sugars such as glucose and xylose inhibit the microbial catalyst performance during fermentation to ethanol. These inhibitors include weak acids (such as acetic acid), aldehydes (for example, furfural), and lignin degradation products (such as vanillin) [1]. Acetic acid, liberated from hemicelluloses during biomass deconstruction, is one of the more dominant inhibitors due to its high concentration in lignocellulosic hydrolysates and its toxic effect on proton gradient homeostasis as a weak acid [2,3]. The development of robust microbial catalysts capable of maintaining high productivity in the presence of acetate and other inhibitors is crucial for commercialization of biochemical conversion processes for biofuel production, and numerous efforts are being devoted to meeting this goal [3].

Although yeast remains a major microbial biocatalyst for ethanol production, other microorganisms such as *E. coli* and *Zymomonas mobilis* have also received significant attention. *Z. mobilis,* a Gram-negative facultative anaerobic ethanologenic bacterium, has excellent industrial characteristics such as unique anaerobic use of the Entner-Doudoroff (ED) pathway that results in a high ethanol yield per mole of glucose consumed, high specific productivity, high ethanol titers, and notable ethanol tolerance [4-9]. In addition, the availability of genome sequence for multiple cultivars [10-14], operon prediction tools [15], metabolic modeling results [16-19], and strain engineering methods [20-25] accelerates the research progress in *Z. mobilis.* However, wild-type *Z. mobilis* can only utilize glucose, fructose, and sucrose as carbon sources, and cannot utilize pentoses like xylose, which is the second most abundant sugar in pretreated and saccharified biomass slurries. An engineered *Z. mobilis* strain 8b was constructed expressing heterologous genes of *talB*, *tktA*, and *xylAB* for xylose utilization as well as truncating the endogenous lactate dehydrogenase gene *ldh* for improved flux to ethanol [23]. Z. *mobilis* 8b is more sensitive to acetate when grown in xylose. The IC50 value (chemical concentration inhibiting 50% cell growth) of acetate when *Z. mobilis* 8b is grown in xylose is 50 mM, compared to the value of 210 mM when glucose is used as the carbon source [1]. The concentration of acetate in a typical hydrolysate prepared from pretreated corn stover at 20% solids loading is about 82 mM, which will completely inhibit cell growth on xylose.

Despite advances in engineering strains of *Z. mobilis* for pentose utilization [23,26-28], co-utilization of glucose and pentoses remains problematic, especially in the presence of inhibitory compounds such as acetate and furfural, and more work will be needed to achieve the high overall ethanol yields required for a commercial process [29-33]*.* Furthermore, despite recent systems biology studies undertaken to unravel the inhibitor tolerance mechanism of *Z. mobilis* for end-product ethanol [15,34], the single inhibitor acetate [35,36] or furfural [37], as well as the comprehensive hydrolysate toxic compounds [24], no transcriptomic study has yet been conducted focusing on the effect and interaction of pretreatment inhibitors and carbon source. This work addresses that knowledge gap and will provide insights on the development of a robust *Z. mobilis* with high pentose utilization efficiency for economic biofuel (both ethanol and advanced hydrocarbon) production.

## Results

### Physiological response to acetate and different sugars

**Figure 1:**
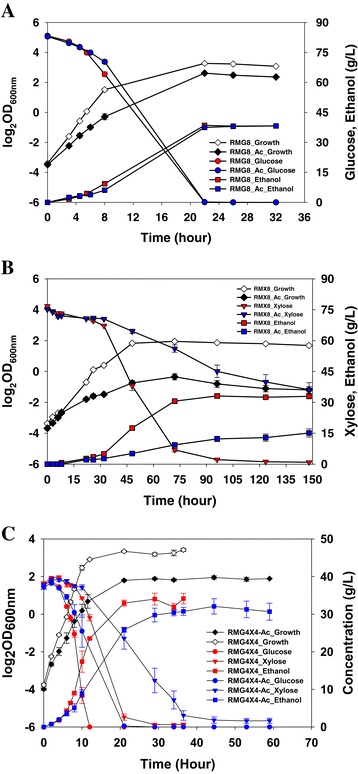
**Cell growth, substrate sugar utilization, and ethanol production in RMG8 (A), RMX8 (B), and RMG4X4 (C) media with and without the supplementation of NH**_**4**_ **OAc.**

As we noted in a previous report for *Z. mobilis* ZM4 [35], the presence of acetate negatively affected cellular growth and ethanol production in *Z. mobilis* fermentations. This is especially true with xylose as the carbon source (Figure 1). The maximum biomass of *Z. mobilis* in terms of OD_600nm_ value in different media (RMG, RMX, or RMGX) without exogenous acetate supplementation was lower than that in media with acetate supplementation, delaying the time to reach the stationary phase and maximal ethanol titers (Figure 1). Correspondingly, the growth rates (h^-1^) in cultures grown in the absence of acetate (RMG or RMX) were at least 50% higher than the growth rates in acetate-supplemented cultures (RMGAc or RMXAc): 0.43 ± 0.01 and 0.093 ± 0.003 in RMG and RMX compared to 0.28 ± 0.021 and 0.056 ± 0.002 in RMGAc or RMXAc, respectively. The sugar source had a dramatic impact on cellular growth; the growth rate of *Z. mobilis* 8b dropped sharply from 0.43 when using glucose to 0.093 when xylose was used as the sole carbon source. The supplementation of glucose restored the cellular growth, and the growth rates of *Z. mobilis* 8b in mixed sugars of glucose and xylose (RMGX and RMGXAc) were same as those in RMG and RMGAc (Figure 1).

Sugar utilization and ethanol production were monitored by HPLC (Figure 1). The elimination of lactate production is consistent with the lactate dehydrogenase gene deletion in the 8b background [23]. Corresponding to the decreased growth, *Z. mobilis* 8b consumed sugar more slowly with acetate supplementation. Ethanol production corresponded to the substrate (glucose or xylose) consumption in all conditions studied in this work; the ethanol was produced gradually as the sugar was consumed and reached its maximum when the glucose or xylose was used up (Figure 1).

### Transcriptomic profiling of *Z. mobilis* in response to acetate using different carbon sources

#### New array design and microarray experiment

To reflect the improved genome annotation [38], we designed a new NimbleGen high-density 12-plex microarray to investigate all genetic features including the gene coding region (CDS) and intergenic regions. The new array contains 146-K features for both expression and tiling arrays covering both the genome and five plasmids as well as heterologous genes (*talB*, *tktA*, *araBAD*, and *xylAB*) engineered into *Z. mobilis* for xylose and arabinose metabolism [26,27].

Samples were taken at exponential phase for the single sugar (RMG or RMX) fermentation experiment, and at different post-inoculation time points of exponential, transition, and stationary phases for mixed sugar (RMGX) fermentation (Figure 1). The time point between the end of the exponential phase and the beginning of the stationary phase was selected as the transition phase. Gene expression profiles were analyzed using a NimbleGen high-density expression array as described previously [35]. In addition to conditions using a sole carbon source of glucose or xylose only, the acetate effect on 8b during mixed sugar RMGX fermentation was also investigated to better model a real production situation using pretreated and saccharified biomass slurries containing both glucose and xylose. In total, 36 arrays were used (Additional file 1: Table S1), and two types of analyses were performed to investigate the effect of acetate on 8b under a variety of conditions: 1) a comparison of the effects of acetate with media (RMG, RMX, or RMGX) and growth phase (log, transition, or stationary) held constant; 2) a comparison of the effects of growth phase (log, transition, or stationary) within different media (RMG, RMX, or RMGX) with or without acetate supplementation.

### Array data quality and qRT-PCR confirmation

The quality of data was checked with good correlation (Additional file 2: Figure S1) before the statistical analyses. The microarray data were also assessed using several approaches, and the quality was found to be high. The correlation coefficients for microarray data from biological replicates were good, with r > 0.9 for each comparison (Additional file 2: Figure S1A, B). In addition, the microarray data of biological replicates were also grouped together closely based on hierarchical clustering and principal components analyses (Additional file 2: Figure S1C, D), and the data can be grouped or separated as individual time points or by treatment.

Moreover, our newly designed microarray contains features of engineered genes such as *talB*, *tktA*, *araBAD*, and *xylAB. Z. mobilis* 8b contains *talB*, *tktA*, and *xylAB* but not *araBAD.* The array data are consistent with our strain background with the *talB*, *tktA*, and *xylAB* genes expressed but not *araBAD* (Additional file 2: Figure S2). Because our array datasets are derived from a high-density tiling array containing multiple probes for each gene, we may obtain information of post-transcriptional regulation through the transcriptomic study. Our results showed that the probe intensities for these engineered genes in different gene locations were different and the probes adjacent to the 5-prime region of these genes had higher expression intensity than those in the C-terminal region (Additional file 2: Figure S2).

Furthermore, gene expression profiles in terms of probe intensity can be overlaid across the whole genome to identify whether there are expression differences in the genome. In this study, most chromosomal regions were expressed evenly under different conditions. However, some regions had relatively higher or lower abundance than the average probe intensity under different conditions (for example, single sugar of RMG and RMX with and without the supplementation of exogenous acetate at exponential phase), which can help visualize the genome regions with different expression patterns and give an overview of gene expression profiles under different conditions (Additional file 2: Figure S3). On the contrary, only small sporadic regions in the plasmids had an expression intensity higher than the background signal and most areas had no expression (Additional file 2: Figure S3). Of the five plasmids reported in *Z. mobilis* [38], sequencing reads were only mapped to portions of three of them, calling for further study to investigate the plasmid profile and the plasmid stability of *Z. mobilis* after metabolic engineering and the role of plasmids in *Z. mobilis* physiology and metabolic networks.

Finally, ten genes from arrays with different expression values were picked for qPCR using a Bio-Rad CFX96 instrument and the Roche qPCR kit. Genes with significant array *P*-values in each comparison were selected, and the correlation between array and qPCR was plotted. The results indicate that our array had good quality with relatively high correlations between qRT-PCR and array results as in our previous reports [15,35,36,39], although the correlation between RMGAc and RMG is relatively weak with a squared correlation coefficient value of 0.79 (Additional file 2: Figure S4).

### Transcriptomic analysis of the effect of acetate and carbon source

#### Overview of acetate and sugar effect

Analysis of variance (ANOVA) analyses using JMP Genomics (SAS Inc., Cary, NC) were carried out using sugar and acetate treatment as variables, and the results indicated that acetate had a more dramatic impact on the transcriptome of 8b in RMG than that of cells grown in RMX, and that the difference between carbon sources is more dramatic than the effect caused by acetate treatment (Additional file 2: Figure S5; Table 1; Additional file 3: Table S2). The overview of significantly differentially expressed genes is shown in Additional file 2: Figure S5. The number of differentially expressed genes in response to acetate or carbon source is summarized in Table 1, and the detailed gene expression information is listed in Additional file 3: Table S2 (S2A for the single sugar experiment and S2B for the mixed sugar array study). With acetate treatment in RMG, only 148 genetic features (including both genes and intergenic region features) were differentially expressed, with 30 genes upregulated and 43 downregulated (Table 1, Additional file 3: Table S2-2). When xylose was used as the sole carbon source, 369 genetic features were differentially expressed compared to that using glucose as the sole carbon source including 113 genes upregulated and 128 genes downregulated (Table 1, Additional file 3: Table S2-4). The RMGX cultures grew much better than the RMX cultures (Figure 1) and, correspondingly, fewer genetic features were differentially expressed in the mixed sugar condition in the exponential phase, with only 28 genes upregulated and 17 downregulated compared to samples taken from the RMG medium (Table 1, Additional file 3: Table S2-17).

**Table 1:** **Summary of significantly differentially expressed genes in response to acetate or carbon sources**

	**cGene**	**cIGR**	**pGene**	**pIGR**	**Total**	**SUM**	**Note**
**Acetate regulated genetic features**							
Gluc_Ac_UP	30	62	3	7	102	148	Acetate regulated genes with glucose as carbon source, Additional file 3: **Table S2-2**.
Gluc_Ac_DOWN	43	3	0	0	46		
Xyl_Ac_UP	18	0	21	8	47	76	Acetate regulated genes with xylose as carbon source, Additional file 3: **Table S2-3**.
Xyl_Ac_DOWN	26	2	1	0	29		
GX_Log_Ac_UP	60	23	1	0	84	154	Acetate regulated genes with mixed glucose and xylose, Additional file 3: **Table S2-11**.
GX_Log_Ac_DOWN	44	26	0	0	70		
**Carbon-source regulated genetic features**							
Xyl/Gluc_UP	113	113	2	5	233	369	Xylose regulated genes compared to glucose, Additional file 3: **Table S2-4**.
Xyl/Gluc_DOWN	128	4	4	0	136		
Xyl/Gluc_Ac_UP	40	2	0	0	42	112	Xylose regulated genes compared to glucose in the presence of acetate, Additional file 3: **Table S2-5**.
Xyl/Gluc_Ac_DOWN	35	33	2	0	70		
GX/Gluc_UP	28	15	2	0	45	73	Mixed sugar (xylose and glucose) regulated genes compared to glucose, Additional file 3: **Table S2-17**.
GX/Gluc_DOWN	17	7	4	0	28		
GX/Gluc_Ac_UP	67	19	1	0	87	151	Mixed sugar (xylose and glucose) regulated genes compared to glucose only in the presence of acetate, Additional file 3: **Table S2-18**.
GX/Gluc_Ac_DOWN	33	24	6	1	64		

cGene: number of chromosomal genes, cIGR: number of chromosomal intergenic regions, pGene: number of plasmid genes, pIGR: number of plasmid intergenic regions. Total: Total number of genetic features including cGene, cIGR, pGene, and pIGR for either upregulated or downregulated genetic features. SUM: the sum of total upregulated and downregulated genetic features.

In addition, when acetate was supplemented into the medium, fewer genetic features were significantly differentially expressed when xylose was used as the sole carbon source than when glucose was used (76 versus 148) (Table 1, Additional file 3: Table S2-2, S2-3). This result further indicates that the utilization of the non-native sugar xylose in the absence of glucose presents a stressful environment for the cells, and the addition of acetate into RMX only causes an incremental transcriptional change since common stress-responsive genes were already expressed in the stressful xylose environment. Alternatively, cells could face dramatic perturbation when shifting from the favorable growth condition using glucose to other stressful conditions (such as xylose utilization or acetate resistance), and more transcriptional regulation is required for cells to adapt to the stress.

### Time-course microarray investigation of the mixed sugar fermentation

**Figure 2:**
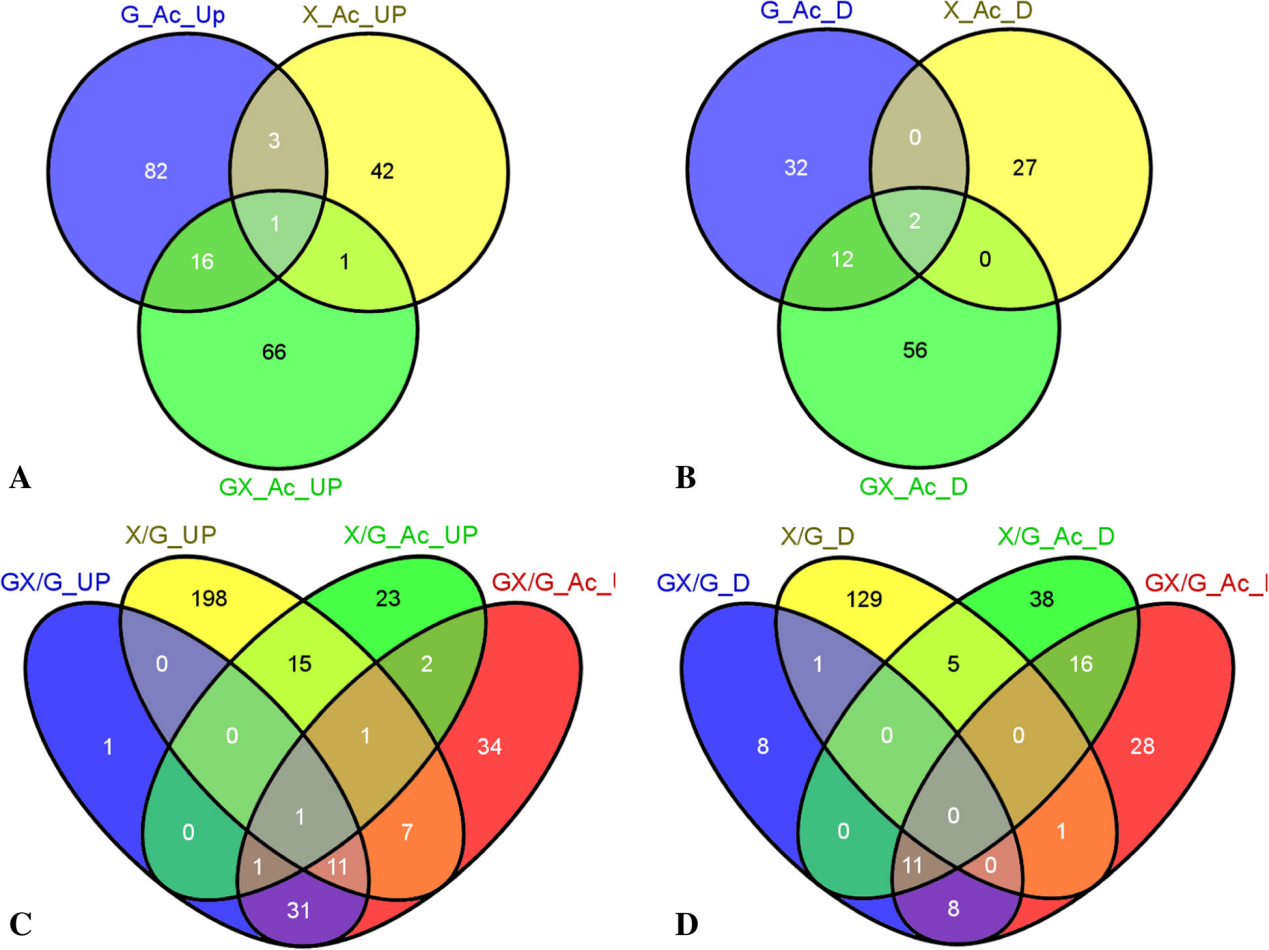
**Venn diagrams of common genetic features shared by shock stress responses in RMG8, RMX8, and RMG4X4. A**: the acetate upregulated features with at least two-fold increase and shared with those upregulated in single sugar of RMG **(G_Ac_UP)** or RMX **(X_Ac_UP)**, and mixed sugar of RMGX **(GX_Ac_UP)**; **B**: the acetate downregulated features with at least two-fold decrease and shared with those downregulated in single sugar of RMG **(G_Ac_D)** or RMX **(X_Ac_D)**, and mixed sugar of RMGX **(GX_Ac_D)**; **C**: genetic features upregulated in xylose RMX **(X/G_UP)** or mixed sugar of RMGX **(GX/G_UP)** compared to that in RMG with at least two-fold increase without acetate supplementation, **X/G_Ac_UP** and **GX/G_Ac_UP** for those with acetate supplementation; **D**: genetic features downregulated in xylose RMX **(X/G_D)** or mixed sugar of RMGX **(GX/G_D)** compared to that in RMG with at least two-fold increase without acetate supplementation, **X/G_Ac_D** and **GX/G_Ac_D** for those with acetate supplementation.

In addition to the comparisons of the *Z. mobilis* 8b transcriptional profiles in RMG, RMX, or RMGX in the absence and presence of acetate, we also investigated the effect of acetate on the gene expression profiles of 8b at the different phases, log, transition, and stationary, in mixed sugar fermentation (Additional file 2: Figure S5, Additional file 3: Table S2-8 to S2-16). In the exponential phase, the effect of mixed sugars on gene expression in the presence of acetate is more like that of glucose than that of xylose with more differentially expressed genetic features (154 features, Table 1, Additional file 3: Table S2-11). In addition, there were more common differentially expressed genetic features shared between mixed sugar and glucose than between mixed sugar and xylose (Figure 2). This suggests that the supplementation of glucose into xylose can alleviate the stress caused by xylose and favor cellular metabolism and growth, which is consistent with the growth, sugar utilization, and ethanol production profiles (Figure 1, Additional file 4: Table S3).

The subsets of expressed genes with at least two-fold changes were used for their potential interactions using the STRING 9.05 precomputed database [40], and the results indicated that acetate upregulated genes in mixed sugar fermentation had better interactions than those of the downregulated ones (Additional file 2: Figure S6). Similar to the individual sugar fermentation, the upregulated genes in the mixed sugar fermentation also included genes encoding cysteine synthase (Additional file 2: Figure S6A). The downregulated ones are also similar to those in individual sugar fermentation, which are involved in biosynthesis (for example, ribosomal proteins), flagellar biosynthesis, and glycolysis (Additional file 2: Figure S6B).

However, we need to point out that these genes may be related to growth phase and may not be acetate specific. For example, dramatic differences were detected when 8b entered the transition phase from the log phase with a large number of genes highly differentially expressed (Additional file 2: Figure S5I, K, M, Additional file 3: Table S2-8, S2-14). The difference between the transition and stationary phases (Additional file 2: Figure S5J, L, N, Additional file 3: Table S2-9, S2-15) was less dramatic than that of the log to transition period. We believe that some genes identified in this study are mainly growth phase dependent rather than acetate or xylose related. This is consistent with our previous studies comparing the impact of stressors (such as oxygen and ethanol) at various stages of the growth phase [15,39]. Caution is needed to identify and interpret stressor-related gene candidates using microarray data from one single time point.

### Transcriptomic study of genes responsive to acetate resistance

We begin our transcriptomic profiling of 8b in response to acetate in RMG, RMX, and RMGX by identifying the common genetic features and those specific to each condition (Figure 2). Briefly, 30 chromosomal genes were upregulated by acetate in RMG (Table 1, Additional file 3: Table S2-2). Six of these are involved in sulfur metabolism including five genes involved in cysteine synthesis (CysC, N, S, K < ZMO003, 4, 7, 0748>, and ZMO0006) and an ABC transport system SsuACB (ZMO1261-3) involved in sulfonate import. The 43 genes downregulated by acetate in RMG were involved in cell motility (for flagellar assembly) and protein synthesis (ribosome component proteins). In the RMX medium, acetate upregulated 18 chromosomal genes (involved in chemotaxis and signal transduction) and downregulated 26 chromosomal genes (involved in biosynthesis and transport) (Table 1, Additional file 3: Table S2-3). However, in the mixed sugar medium RMGX, 60 chromosomal genes were found to be upregulated by acetate, mainly involved in oxidation-reduction reactions and amino acid metabolism. Acetate caused 44 chromosomal genes to be downregulated in RMGX, mainly coding for hypothetical proteins (Table 1, Additional file 3: Table S2-11).

Genes shared under different conditions were investigated, and ZMO1682 is the only upregulated gene responsive to acetate supplementation in all three growth conditions. This gene encodes L-aspartate beta-decarboxylase involved in amino acid metabolism (Figure 2A, Additional file 3: Table S2-2, S2-3, S2-11). Another gene upregulated by acetate in both RMG and RMX but not in RMGX is ZMO1181, which encodes N-formylglutamate amidohydrolase. Only one other gene, ZMO1885 (*ncr*) encoding NADH-flavin oxidoreductase, is upregulated by acetate in RMX and RMGX. In contrast, ten genes are upregulated by acetate in both RMG and RMGX. Three of these are involved in sulfur metabolism: cysteine synthase CysD, I, J (ZMO0005, 8, 9) and individual genes are involved in stress response: Fe-S oxidoreductase (ZMO0022), aldo/keto reductase (Dkg, ZMO1344), and catalase (ZMO0918). Other upregulated genes encode the TonB-dependent receptor (ZMO0031), glucan biosynthesis protein D (ZMO0905), L-sorbose reductase (ZMO1449), and a potential alanine/aspartate transporter (ZMO1681) (Figure 2A, Additional file 3: Table S2-2, S2-3, S2-11).

The two genes downregulated by acetate in RMG, RMX, and RMGX are ZMO0128 and ZMO1522, which are annotated as TonB-dependent receptors (Figure 2B, Additional file 3: Table S2-2, S2-3, S2-11). Nine additional genes were downregulated by acetate in RMG and RMGX. With the exception of ZMO0127 (encoding a nuclease S1), ZMO0130 (*phoC,* encoding an acid phosphatase precursor), ZMO1020 (encoding a decarboxylase 2), and ZMO0412 (encoding a potential multiple antibiotic transporter), the other five genes (ZMO0095, 0110, 0131, 0912, and 1521) encode hypothetical proteins.

Clearly from the Venn diagrams (Figure 2), the genes responsive to acetate in RMX, RMG, or RMGX alone far exceed those shared in two or more media. These results indicate that acetate has little further effect than the stress of growth in xylose compared to growth in glucose or glucose plus xylose. The slightly altered pattern of genes differentially expressed between RMG and RMGX suggests that xylose does exert subtle effects in RMGX (Figure 2, Additional file 3: Table S2-2, S2-11).

### Transcriptomic study of genes responsive to xylose utilization

To investigate the effect of the non-native sugar xylose on 8b gene expression, the transcriptomic profile of 8b in RMG was compared to those of cells grown in RMX (Additional file 3: Table S2-4) or RMGX (Additional file 3: Table S2-17). Only one common gene was identified to be upregulated, ZMO1268, a hypothetical protein containing a truncated MarR domain with very low similarity (Evalue = 2.31e-03), no matter whether the exogenous acetate was present or not.

Since the growth and gene expression profiles in RMGX were similar to those in RMG (Figure 2, Table 1), we focused our subsequent analysis of differentially expressed genes on results from cells grown in RMG and RMX. The utilization of non-native xylose apparently poses a significant metabolic burden to the cells, with 113 genes upregulated and 128 downregulated (Table 1, Additional file 3: Table S2-4), and the gene functions of these differentially expressed genes have much in common with acetate-responsive genes as discussed above as well as with other stress (ethanol or furfural) responsive genes as described in previous reports [34,41]. In the presence of acetate, only 40 genes were upregulated and 35 genes downregulated in RMX compared to RMG (Table 1, Additional file 3: Table S2-5). This indicates that multiple stressors elicit the same basic response.

Indeed, 7 upregulated genes and 16 downregulated ones are common between the acetate-responsive genes in RMG (Additional file 3: Table S2-2) and the xylose-responsive genes without acetate supplementation (RMX versus RMG, Additional file 3: Table S2-4). These common genes may serve as genetic targets for improving microbial tolerance to different stressors. However, we also point out that, despite the commonality, there are many more transcriptional responses unique to the individual stressors. Growth in xylose led to upregulation of 113 genes and downregulation of 128 genes compared to growth in glucose, while only 23 genes were acetate upregulated and 27 were acetate downregulated (Figure 2, Table 1, Additional file 3: Table S2-2, S2-4).

### Genetics confirmation

As noted above, our transcriptomic analysis provided targets for strain improvement to increase acetate tolerance. We constructed chromosomal knockin mutant strains to overexpress the genes that were upregulated to acetate as well as knockout mutants of the upregulated and downregulated genes. We hypothesized that the overexpression of the upregulated genes as well as deletion of the downregulated genes could confer growth advantage in the presence of acetate, while the deletion of the upregulated genes would pose a negative effect. Unfortunately, our knockin strategy was unsuccessful in generating stable mutants, perhaps due to the choice of a constitutive strong promoter in our constructs (data not shown). However, we were successful in constructing three knockout mutants (8b-KO1181, 8b-KO1682, and 8b-KO0128, named for the cognate gene). These mutant strains grew similarly as their parental strain 8b in RMG without exogenous acetate supplementation (data not shown). 8b-KO1181 and 8b-KO1682 were indistinguishable from the control strain, while 8b-KO0128 had a slight advantage in acetate resistance for cultures grown in RMG plus acetate (Additional file 2: Figure S7). A BLAST search of the *Z. mobilis* genome indicated that ZMO1682 and ZMO1181 are homologues of ZMO0342 (*aatA*) and ZMO1395, respectively, and these homologous gene products may compensate for the effect of the deletion. Similarly, the existence of multiple homologues of ZMO0128 in *Z. mobilis* (Additional file 2: Figure S8) may explain why the knockout of ZMO0128 only conferred a slight advantage in acetate tolerance. Further directed knockout mutants to eliminate other homologous genes may boost the effect of acetate tolerance.

## Discussion

### The exogenous acetate stress or non-native xylose utilization shifted redox balance, leading to toxic intermediate xylitol formation and glycolysis inhibition

Glycolysis and fermentation provide not only the necessary metabolic intermediates but also energy forms of ATP and redox NAD(P)H for anabolism, and the enzymes involved in glycolysis are among the most abundant proteins in Z. *mobilis* [15]. The stressful conditions trigger energy conservation and inhibit central metabolism with genes related to stress response being upregulated [15,39]. In addition, the expression levels for genes encoding the glycolytic enzymes were also affected by the stressful condition. For example, our previous study suggests that the transcriptional levels of glycolytic enzymes (such as Gpm, Eno, and Pyk) were reduced in the aerobic culture compared to that of the anaerobic culture. In addition, glycolysis intermediates were accumulated which may be due to the reduced glycolysis efficiency [39]. A similar energy conservation phenomenon under stressful conditions has also been observed for *E. coli* [42,43].

**Figure 3:**
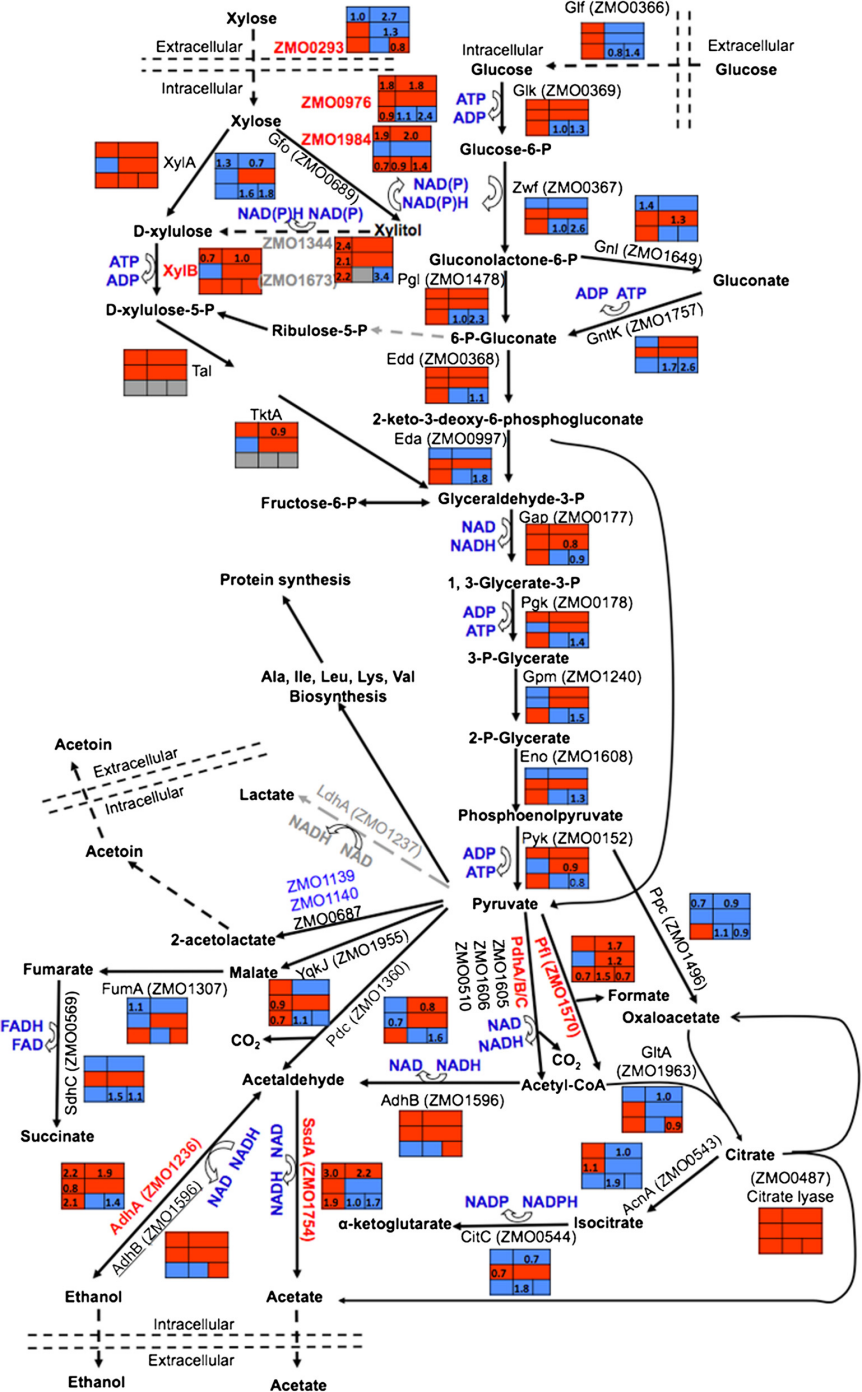
**Metabolic pathway of xylose degradation, glycolysis, and fermentation in** ***Z. mobilis*** **8b strain.** The box adjacent to the enzyme contains gene expression information from Additional file 3: Table S2-1, Table S2-6. The top row is for sugar effect: xylose-responsive genes compared to glucose in log phase without or with the supplementation of exogenous acetate from left to right column respectively, and the middle row is for acetate effect in log phase when exogenous acetate was added into single sugar of glucose or xylose respectively, from left to right column (Additional file 3: Table S2-1). The bottom row is for acetate effect in mixed sugar in log, transition, or stationary phase respectively, from left to right column (Additional file 3: Table S2-6). Absolute values greater than 1.5-fold (number in the box is log_2_-based value) were used, with red-filled box indicating upregulation and blue-filled box downregulation.

To investigate the impact of acetate supplementation and xylose utilization on glycolysis and fermentation, the metabolic pathway including xylose metabolism, glycolysis, and fermentation in the *Z. mobilis* 8b strain was reconstructed, and transcriptomic data were mapped into the pathway for xylose and acetate responsive genes under different conditions (Figure 3). When glucose was used as the only carbon source, the efficient *Z. mobilis* fermentation pathway enzymes Pdc (ZMO1360) and AdhB (ZMO1596) allowed fast ethanol production with minimal xylitol and acetate produced as byproducts (Figure 3, Additional file 4: Table S3). However, when xylose was the only carbon source, the acetate and xylitol levels were much higher than those in the RMG cultures (Additional file 4: Table S3). Comparing cells grown in RMX versus RMG, the *ssdA* (ZMO1754) gene encoding aldehyde dehydrogenase, which catalyzes the conversion of acetaldehyde to acetate, was upregulated eight-fold, and the *adhA* (ZMO1236) gene, encoding an iron-containing group III alcohol dehydrogenase proposed to oxidize ethanol into acetaldehyde [44,45], was also upregulated about four-fold. Similar results were seen when exogenous acetate was added into the medium, which suggests that the acetate accumulation may cause a buildup of the NADH reducing equivalents, leading to redox imbalance (Figure 3).

Excessive amounts of NADH could then drive the reaction of xylose conversion towards the accumulation of xylitol instead of the production of D-xylulose to regenerate NAD for redox balance (Figure 3). As evidence of this hypothesis, there was a high correlation (R^2^ = 0.99) between the xylose consumed and the xylitol accumulated (Additional file 4: Table S3). In addition, ZMO0976, a gene proposed to convert xylose to xylitol [46], was also upregulated consistent with a need to balance the excessive NADH accumulated, which is detrimental to the cells. The knockout of this gene has been proved to help *Z. mobilis* resist acetate toxicity [29]. Furthermore, ZMO1984, a paralogue to ZMO0976, was also upregulated similarly in response to xylose, which may further drive the reaction toward xylitol production (Figure 3). Although the reaction from xylose to xylitol could mitigate the problem of redox imbalance, xylitol is toxic to the cells and causes growth inhibition. In addition, the *gfo* gene encoded by ZMO0689 was reported to carry out the reaction from xylose to xylitol as well [47], and it was downregulated in RMX compared to RMG. Taken together, these observations indicate that the conversion of xylose to xylitol is more complicated than previously thought, as this reaction is involved in multiple responses to metabolic stressors, all leading to a toxic dead-end product of xylose (Figure 3).

Glf (ZMO0366) has been proposed to serve as a xylose transporter in addition to its primary role as a glucose transporter [37]. A major facilitator superfamily (MFS) family sugar transporter ZMO0293 (EC: 1.3.1.74) is homologous to Glf with a high similarity score of 191. ZMO0293 encodes a symporter with 12 transmembrane domains, which also has high similarity to xylose and arabinose symporters of *E. coli* (for example, XylE). ZMO0293 was downregulated when using xylose (RMX) compared to that in RMG in the absence or presence of exogenous acetate. In addition, when xylose was the sole carbon source, ZMO0293 was downregulated further in the presence of acetate (Figure 3). Furthermore, ZMO0293 was downregulated in late phases (transition or stationary) compared to the log phase with or without the acetate supplementation. This is consistent with its xylose downregulation phenomenon, since glucose was completely consumed with xylose present when reaching the transition or stationary phases (Figure 1, Additional file 3: Table S2-1, S2-6, Additional file 4: Table S3). We therefore propose that ZMO0293 may be the major transporter involved in xylose importation, and the downregulation of ZMO0293 in the presence of xylose may explain the poor xylose utilization by *Z. mobilis* and could be a genetic target to improve xylose utilization in *Z. mobilis* (for example, by changing the promoter region to release the transcriptional repression in the presence of xylose)*.*

As described above, acetate significantly inhibits growth in RMX medium. About half of the xylose was still remaining in the media at 148 h post inoculation and the ethanol concentration was only about half that in RMX without acetate supplementation (Figure 3, Additional file 4: Table S3). The addition of acetate into RMX caused the downregulation of ZMO0293, which could reduce xylose uptake in *Z. mobilis.* At the same time, the Pfl gene (ZMO1570) was upregulated, which could drive more pyruvate into the acetate production, causing NADH buildup and more xylitol accumulation. For example, when about half of the xylose (38 g) was consumed, the xylitol concentration was 2.38 g/L in the absence of acetate compared to 3.37 g/L with the supplementation of acetate (Additional file 4: Table S3). Similarly, when the RMGX medium was used, xylitol was higher in the presence of acetate than in its absence (Figure 1, Additional file 4: Table S3). This result indicates that the stress from exogenous acetate can cause a toxic chain reaction by driving carbon flux toward acetate production with excessive NADH accumulation, shifting from complete metabolism of xylose via xylulose to xylitol generation, inhibiting growth and ethanol production (Figure 1).

### Alternative energy metabolism pathways to overcome the energy shortage due to glycolysis inhibition

Previous studies have indicated that, besides energy conservation, microorganisms might bypass the central glycolysis pathway and utilize alternative pathways (such as the nitrogen metabolism in *Clostridium thermocellum* ATCC27405 [48]) for carbon metabolism and energy regeneration to survive the harsh conditions. Additionally, sulfur assimilation was suggested to contribute to *Z. mobilis* ethanol stress response and hydrolysate tolerance [15,24]. In this study, several sulfur assimilation-related genes were also upregulated in RMG medium in the presence of acetate. For example, *cysI, J, K, N* were upregulated, and genes related to glutathione synthesis needed for assembly of Fe-S clusters were also upregulated in a response to acetate. Other general stress response genes were also upregulated in response to acetate in this study including genes encoding proteases (Clp), chaperone proteins (GroEL), and nucleases, which will help repair the damaged proteins and DNA to ensure accurate cellular function. Furthermore, metabolism of these protein and DNA degradation products may also help resolve any energy imbalances.

Moreover, ZMO1181 was upregulated in both RMG and RMX media in the presence of acetate. ZMO1181 is a single gene transcription unit (TU) encoding N-formylglutamate amidohydrolase, which catalyzes the hydrolysis reaction to release amino acids such as alanine from bacterial cell-wall glycopeptides as a substrate for alternative carbon metabolism. All of these results suggest that *Z. mobilis* may bypass the inhibition on central metabolism by external stressful conditions and utilize alternative carbon and energy metabolism pathways to provide energy and metabolic precursors to keep redox balanced and the cell growing under stressful conditions (Figure 3). Additional aspects of energy conservation and generation will be addressed in the following section.

### Amino acid decarboxylase for energy generation

Amino acid decarboxylation has been suggested to help microorganisms resist acid stress. For example, aspartate-alanine antiporters and histidine decarboxylation enzymes help proton motive force (PMF) generation and ATP formation in *Lactobacillus* sp. [49,50]. In addition, four genes involved in amino acid decarboxylation were reported to be associated with acid tolerance of *L. acidophilus* [51]. The consistent upregulation of ZMO1682 in *Z. mobilis* 8b in response to acetate was independent of sugar source of glucose, xylose, or the mixed sugar of RMGX, indicating its critical role in resistance to the weak acid of acetate. ZMO1682 encodes an aspartate aminotransferase and forms an operon with ZMO1681, which encodes a putative aspartate-alanine antiporter. This operon is coexpressed under ethanol and salt conditions [15] as well as the acetate condition in this study. This operon likely responds to the stress such as xylose utilization and acetate supplementation in the early stage, and the upregulation of this operon may help *Z. mobilis* generate PMF and form ATP for acetate tolerance. During acetate stress conditions, genes encoding proteases and chaperone proteins were upregulated (Additional file 3: Table S2-2, S2-3, S2-11), which may help repair and at the same time degrade these damaged proteins to release the amino acids as substrates for this decarboxylation reaction and resist the acetate stress.

### The dilemma of sugar transport on substrate importation and energy conservation

Glf (ZMO0366, EC: 1.3.1.74) was regarded as the major sugar transporter and reported to be responsive for the transport of non-native sugars such as xylose [37]. In this study, *glf* was only downregulated from the log phase to transition and stationary phases in RMGX in the presence of exogenous acetate. Its expression was similar in RMG or RMX with or without acetate supplementation at log phase (Additional file 3: Table S2). However, *Z. mobilis* growth in RMG or RMX was dramatically different (Figure 1). Besides the impact of toxic xylitol accumulation and redox imbalance as discussed above, other genetic elements may also be responsive for these physiology differences of *Z. mobilis*.

As discussed briefly above, the expression of XylE homologous gene ZMO0293 was growth-phase dependent in the mixed sugar fermentation and downregulated in late growth phases when glucose was consumed. The expression of ZMO0293 was also downregulated by xylose (Additional file 3: Table S2-4, S2-5). This is different from its *E. coli* XylE symporter, which is repressed by glucose but induced by xylose. The expression of ZMO0293 was also downregulated by acetate in RMX medium (Additional file 3: Table S2-3), which indicates that the existence of acetate in the media may reduce xylose transport, thus interfering with carbon metabolism. Conceivably, the importation of both sugar and proton by this kind of MFS family symporter would change the PMF gradient and further acidify the cell, especially in the presence of the weak acid acetate or in stationary phase when metabolism decreases and the cells are less capable of pumping protons out for energy regeneration. Although these gene expression patterns and cell physiology differences indicate the involvement of sugar transport in stress response, further work is needed to systematically investigate the transport kinetics and substrate specificity of Glf and ZMO0293 under similar growth conditions to those used for this study. Upregulation of another transporter ZMO1452 (AraJ) with low similarity to Glf from log to transition phase (Additional file 3: Table S2-14) and its downregulation from transition to stationary (Additional file 3: Table S2-15) as well as the downregulation in the presence of acetate in stationary phase (Additional file 3: Table S2-6) demonstrate the importance and complexity of sugar transport in adapting to different environmental conditions.

### TonB-dependent transporters and energy conservation

TonB-dependent receptors are a family of beta-barrel proteins in the Gram-negative bacteria outer membrane, which can transport substrates of iron chelators (siderophores), oligosaccharides, and polypeptides against the concentration gradient with two other components of a periplasma-facing TonB protein and a plasma membrane localized machinery (ExbBD). In *E. coli*, the TonB protein interacts with outer membrane receptor proteins that carry out high-affinity binding and energy-dependent uptake of specific substrates into the periplasmic space [2]. In this study, two genes (ZMO0128 and ZMO1522) annotated as TonB-dependent receptors were consistently downregulated under different conditions. Potentially, ZMO0128 is a paralogue to four other *Z. mobilis* genes annotated as TonB-dependent receptors (ZMO0789, ZMO1463, ZMO1568, and ZMO1694) with a close relationship (Additional file 2: Figure S8). ZMO1522 is not similar to ZMO0128 and distinct from other genes annotated as TonB-dependent receptors such as ZMO0171, ZMO0979, ZMO1040, and ZMO1260. Six other TonB-dependent receptors are more similar to ZMO0128 than to ZMO1522 (Additional file 2: Figure S8). The downregulation of these two TonB-dependent receptors may therefore reduce energy needs for substrate uptake and reserve the energy for stress responses. The ZMO0128 knockout mutant did have a slight advantage over the wild-type strain for acetate tolerance (Additional file 2: Figure S7). However, further investigation is needed to identify the substrates that these TonB-dependent receptors are binding to, and the relationship between the substrate uptake repression and energy conservation.

## Conclusions

The physiological responses and transcriptomic profiles of an engineered xylose-utilizing *Z. mobilis* strain 8b to acetate stress using glucose or xylose individually or in mixed sugar fermentations were investigated. The non-native sugar xylose poses a stress for 8b, and the supplementation of acetate impedes the cell growth of 8b, resulting in slow substrate utilization and ethanol production. The transcriptomic profiles indicate that the molecular responses of 8b to xylose and acetate are dynamic and complicated. The utilization of the non-native sugar, xylose, elicits a more dramatic effect on transcriptional regulation than the inhibitor, acetate. Our study also suggests that stressful conditions such as growth in xylose in the presence and absence of acetate cause a redox imbalance. As the cells adjust metabolism to address redox imbalance, carbon flux may shift towards the accumulation of a toxic intermediate such as xylitol. In addition, *Z. mobilis* appears to manage its energy balance through the reduction of energy-consuming reactions such as expression of genes encoding efflux pumps and substrate transporters as well as energy regeneration through alternative carbon and energy pathways such as amino acid decarboxylation. Several target gene candidates based on the transcriptomic results were selected for genetic manipulation, and a TonB-dependent receptor knockout mutant was shown to convey acetate tolerance. Our study has provided insights into the molecular responses of the model ethanologenic bacterium *Z. mobilis* to the inhibitor acetate in different sugar sources, which will help further strain metabolic engineering efforts for better xylose utilization and acetate tolerance (for example, deletion of ZMO0293 for improved xylose transportation and utilization of the substrate channeling technique to direct the substrate xylose into the pentose phosphate pathway instead of toxic intermediate xylitol accumulation).

## Materials and methods

### Bacterial strain and growth conditions

*Z. mobilis* 8b was revived from frozen glycerol stocks for about 6 to 8 h in 10 mL RM (10 g/L yeast extract, 2 g/L KH_2_PO_4_) + 2% glucose at 33°C prior to inoculating the seed cultures overnight in RM + 5% glucose (RMG5) using shake flasks filled to 80% capacity at 33°C at 120 rpm. When the glucose concentration reached approximately 20 to 40 g/L, the cells were spun down at 3,840 × g for 10 min at room temperature (RT) and resuspended in either RMG (8% glucose), RMX (8% xylose), or RMGX (4% glucose, 4% xylose) at a ten-fold concentration and used as inocula for fermentation studies.

### Controlled batch fermentations

Fermentations were performed in BioStat-Q Plus fermentors (Sartorius Stedim Biotech, France) at 300-mL working volumes using *Z. mobilis* 8b. RMG, RMX, or RMGX media were used for fermentation. The fermentors were inoculated at an OD_600nm_ unit of 0.1 approximately. The fermentation was conducted at 30°C with an agitation rate of 150 rpm. The pH control set point was maintained at 6.0 by automatic titration with 2 N KOH. Samples were harvested during fermentation at different time points for transcriptomic and HPLC analyses as described previously [39]. The time point between the end of the exponential phase and the beginning of the stationary phase was selected as the transition phase. Specifically for the mixed sugar fermentation with multiple sampling time points, the exponential, transition, and stationary time points without acetate supplementation are 7, 12, and 36.5 h post inoculation, whereas the exponential, transition, and stationary time points with acetate supplementation are 11.5, 21, and 59 h post inoculation.

### Bioscreen C high-throughput toxicity assay

The high-throughput Bioscreen C assay was carried out as reported previously [1,52]. Briefly, *Z. mobilis* cells were revived from overnight culture with OD_600nm_ adjusted to 1.5, and 10 μL seed culture was added into 290 μL media with an initial OD_600nm_ of about 0.05. Growth was then monitored using a Bioscreen C instrument (GrowthCurves, Piscataway, NJ, USA) with three technical replicates. The experiments were repeated at least two times.

### Extracellular metabolite analysis with HPLC

HPLC analysis was used for the measurements of the extracellular metabolite concentration of glucose, xylose, acetate, ethanol, xylitol, glycerol, and lactate in 0.2 μm-filtered samples taken at different time points during fermentation. Soluble fermentation products were identified by comparison with retention times and peak areas of corresponding standards as described previously [15,35,39,53].

### RNA extraction and ds-cDNA synthesis

Total RNA was isolated from the cell pellet resuspended in TRIzol reagent (Invitrogen, Carlsbad, CA) as described previously [39]. Each total RNA preparation was treated with RNase-free DNase I (Ambion, Austin, TX) to digest residual genomic DNA and subsequently purified using the RNeasy Mini Kit (Qiagen, Valencia, CA). Total cellular RNA was quantified with a NanoDrop ND-1000 spectrophotometer (NanoDrop Technologies, Wilmington, DE) and RNA quality was assessed with an Agilent Bioanalyzer (Agilent, Santa Clara, CA). Purified RNA of high quality was used as the template to generate ds-cDNA using Invitrogen ds-cDNA synthesis kit (Invitrogen, CA).

### Microarray sample labeling, hybridization, scan, and statistical analysis of array data

Ds-cDNA was labeled, hybridized, washed, and scanned following the NimbleGen protocols. Hybridizations were conducted using a 12-bay hybridization station (BioMicro Systems Inc., Salt Lake City, UT), the arrays were dried using a Maui wash system (BioMicro Systems Inc.) and scanned with a SureScan high-resolution DNA microarray scanner (Agilent Technologies, CA), and the images were quantified using NimbleScan software (Roche NimbleGen, IN). Statistical analyses were done with JMP Genomics 5.1 software (SAS Institute, Cary, NC) as described previously [35,39]. Briefly, raw data of probe fluorescence intensity were log_2_ transformed and imported into JMP Genomics for analysis. A distribution analysis and data correlation analysis were conducted as a quality control step. The overlaid kernel density estimates derived from the distribution analysis allowed the visualization of sources of variation based on strain and treatment, as well as variation attributed to technical factors, such as array. The data were subsequently normalized using the LOESS Normalization algorithm within JMP Genomics. An analysis of variance (ANOVA) was performed to determine differential expression levels between conditions and time points using the false discovery rate (FDR) testing method (*P* < 0.05). The significantly differentially expressed gene lists for different comparisons were generated and outputted as Excel sheets. Microarray studies have been submitted to NCBI GEO database with the accession number GSE57553. The interactions among differentially expressed genes were investigated using the STRING 9.05 precomputed database [40] available at http://string.embl.de/.

### Chromosomal knockout deletion constructs

The Gibson Assembly kit (New England BioLabs, Ipswich, MA) was used to assemble upstream and downstream fragments (about 1.2 kb each) of the target deletion gene with the spectinomycin gene *aadA* in the middle. The assembled product was used as a template for PCR amplification. The purified PCR product was directly electroporated into the *Z. mobilis.* Transformants appearing on RMG plates with the supplementation of spectinomycin were screened using colony PCR. Colonies with correct PCR product sizes were selected as deletion candidates after sequencing confirmation using the Sanger sequencing service from GenScript (Piscataway, NJ, USA).

## Additional files

Additional file 1: Table S1. Information for array data used for acetate stress response transcriptomic profiling. F: fermentor number. Additional file 2: Figure S1. The quality control analyses of microarray data by JMP Genomics. **Figure S2.** Example of the recombinant gene expression for *Z. mobilis* in RMG at exponential phase for each probe with two biological replicates listed together for each gene. **Figure S3.** Transcriptomic profiles of *Z. mobilis* gDNA and plasmid genes in single sugar of RMG8 or RMX8 with and without the supplementation of exogenous ammonium acetate based on tiling array data. **Figure S4.** Correlations between qRT-PCR and microarray. **Figure S5.** The ANOVA modeling of acetate shock response microarray data using JMP Genomics. **Figure S6.** Interactions among acetate upregulated genes and downregulated genes for *Z. mobilis* 8b grown in mixed sugar of RMG4X4 with at least twofold significant increase (data from Table S2-7) using the STRING precomputed protein-interaction database. **Figure S7.** Bioscreen C result of wild-type 8b and ZMO0128 knockout mutant 8b-KO0128 grown in RMG8 with the supplementation of ammonium acetate (15 g/L). **Figure S8.** Relationship among TonB-dependent receptors in *Z. mobilis*. Additional file 3: Table S2. Lists of significantly differentially expressed genes for single sugar experiment (S2A including 5 Excel sheets from S2-1 to S2-5) and for mixed sugar array study (S2B including 13 Excel sheets from S2-6 to S2-18). Additional file 4: Table S3. HPLC data of glucose, xylose, xylitol, glycerol, acetic acid, and ethanol (g/L) when 8b grown in RMG8, RMX8, and RMG4X4 media with and without the supplementation of NH_4_OAc at different time points.

## Abbreviations

CDS: gene coding region
ED: Entner-Doudoroff pathway
HPLC: high-performance liquid chromatography
IC50: chemical concentration inhibiting 50% cell growth
MFS: major facilitator superfamily transporter
PMF: proton motive force
TU: transcription unit
