# Supplementary material for: Insights into acetate toxicity in Zymomonas mobilis 8b using different substrates
Source: Biotechnol Biofuels. 2014 Sep 30;7:140. doi: 10.1186/s13068-014-0140-8 (PMC4189746; doi:10.1186/s13068-014-0140-8)
Supplement: Additional file 1: Table S1. — Information for array data used for acetate stress response transcriptomic profiling. F: fermentor number. [file 13068_2014_140_MOESM1_ESM.docx]

**Table S1. The information of array data used for acetate stress response transcriptomic profiling. F:** fermentor number.

| **Sample Description** | **Sugar** | **Treatment** | **Phase** | **F** |
| --- | --- | --- | --- | --- |
| HT09_F1_log_2 | Glucose and xylose | None | log | F1 |
| HT09_F2_log_2 | Glucose and xylose | None | log | F2 |
| HT09_F3_log_2 | Glucose and xylose | None | log | F3 |
| HT09_F1_Transition_2 | Glucose and xylose | None | Transition | F1 |
| HT09_F2_Transition_1 | Glucose and xylose | None | Transition | F2 |
| HT09_F3_Transition_1 | Glucose and xylose | None | Transition | F3 |
| HT09_F1_Stationary_2 | Glucose and xylose | None | Stationary | F1 |
| HT09_F2_Stationary_1 | Glucose and xylose | None | Stationary | F2 |
| HT09_F3_Stationary_2 | Glucose and xylose | None | Stationary | F3 |
| HT09_F4_log_1 | Glucose and xylose | 10g/L NH_4_OAc | log | F4 |
| HT09_F5_log_2 | Glucose and xylose | 10g/L NH_4_OAc | log | F5 |
| HT09_F6_log_1 | Glucose and xylose | 10g/L NH_4_OAc | log | F6 |
| HT09_F4_Transition_1 | Glucose and xylose | 10g/L NH_4_OAc | Transition | F4 |
| HT09_F5_Transition_1 | Glucose and xylose | 10g/L NH_4_OAc | Transition | F5 |
| HT09_F6_Transition_2 | Glucose and xylose | 10g/L NH_4_OAc | Transition | F6 |
| HT09_F4_Stationary_2 | Glucose and xylose | 10g/L NH_4_OAc | Stationary | F4 |
| HT09_F5_Stationary_2 | Glucose and xylose | 10g/L NH_4_OAc | Stationary | F5 |
| HT09_F6_Stationary_2 | Glucose and xylose | 10g/L NH_4_OAc | Stationary | F6 |
| HT08_F1_T0_1_2 | Glucose | None | log | F1 |
| HT08_F2_T0_1_2 | Glucose | None | log | F2 |
| HT08_F3_T0_1_2 | Glucose | None | log | F3 |
| HT08_F7_T0_F | Xylose | None | log | F7 |
| HT08_F8_T0_F | Xylose | None | log | F8 |
| HT08_F9_T0_F | Xylose | None | log | F9 |
| Z.Mobilis8b_Glucose | Glucose | None | log | F1 |
| Z.Mobilis8b_Glucose | Glucose | None | log | F2 |
| Z.Mobilis8b_Glucose | Glucose | None | log | F3 |
| Z.Mobilis8b_Glucose_Acetate | Glucose | 10g/L NH_4_OAc | log | F4 |
| Z.Mobilis8b_Glucose_Acetate | Glucose | 10g/L NH_4_OAc | log | F5 |
| Z.Mobilis8b_Glucose_Acetate | Glucose | 10g/L NH_4_OAc | log | F6 |
| Z.Mobilis8b_Xylose | Xylose | None | log | F7 |
| Z.Mobilis8b_Xylose | Xylose | None | log | F8 |
| Z.Mobilis8b_Xylose | Xylose | None | log | F9 |
| Z.Mobilis8b_Xylose_Acetate | Xylose | 3.5g/L NH_4_OAc | log | F10 |
| Z.Mobilis8b_Xylose_Acetate | Xylose | 3.5g/L NH_4_OAc | log | F11 |
| Z.Mobilis8b_Xylose_Acetate | Xylose | 3.5g/L NH_4_OAc | log | F12 |
