# Supplementary material for: Insights into acetate toxicity in Zymomonas mobilis 8b using different substrates
Source: Biotechnol Biofuels. 2014 Sep 30;7:140. doi: 10.1186/s13068-014-0140-8 (PMC4189746; doi:10.1186/s13068-014-0140-8)
Supplement: Additional file 2: Figure S1. — The quality control analyses of microarray data by JMP Genomics. Figure S2. Example of the recombinant gene expression for Z. mobilis in RMG at exponential phase for each probe with two biological replicates listed together for each gene. Figure S3. Transcriptomic profiles of Z. mobilis gDNA and plasmid genes in single sugar of RMG8 or RMX8 with and without the supplementation of exogenous ammonium acetate based on tiling array data. Figure S4. Correlations between qRT-PCR and microarray. Figure S5. The ANOVA modeling of acetate shock response microarray data using JMP Genomics. Figure S6. Interactions among acetate upregulated genes and downregulated genes for Z. mobilis 8b grown in mixed sugar of RMG4X4 with at least twofold significant increase (data from Table S2-7) using the STRING precomputed protein-interaction database. Figure S7. Bioscreen C result of wild-type 8b and ZMO0128 knockout mutant 8b-KO0128 grown in RMG8 with the supplementation of ammonium acetate (15 g/L). Figure S8. Relationship among TonB-dependent receptors in Z. mobilis. [file 13068_2014_140_MOESM2_ESM.docx]

**A**


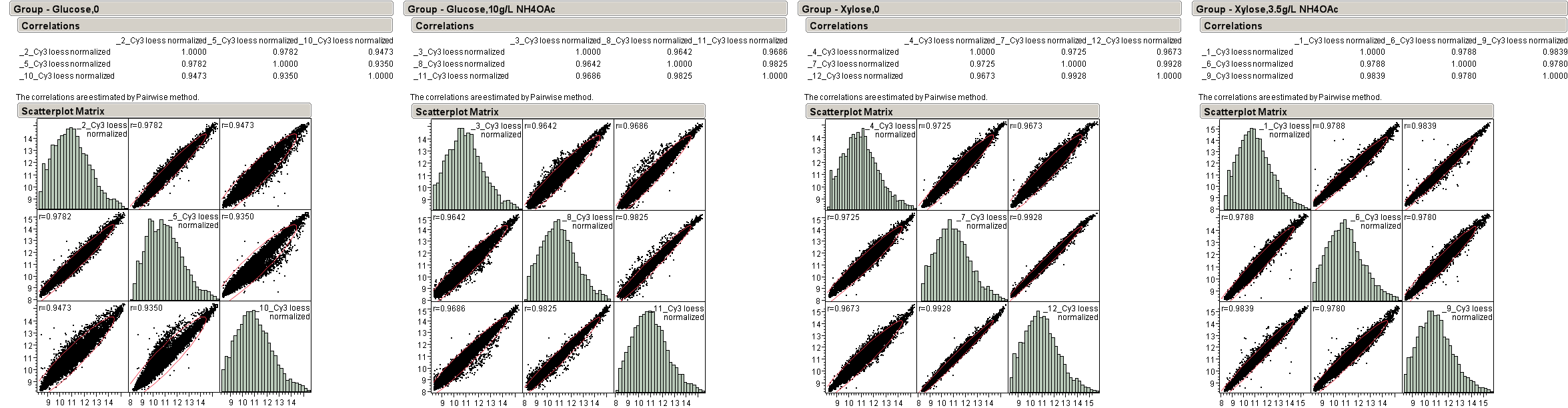

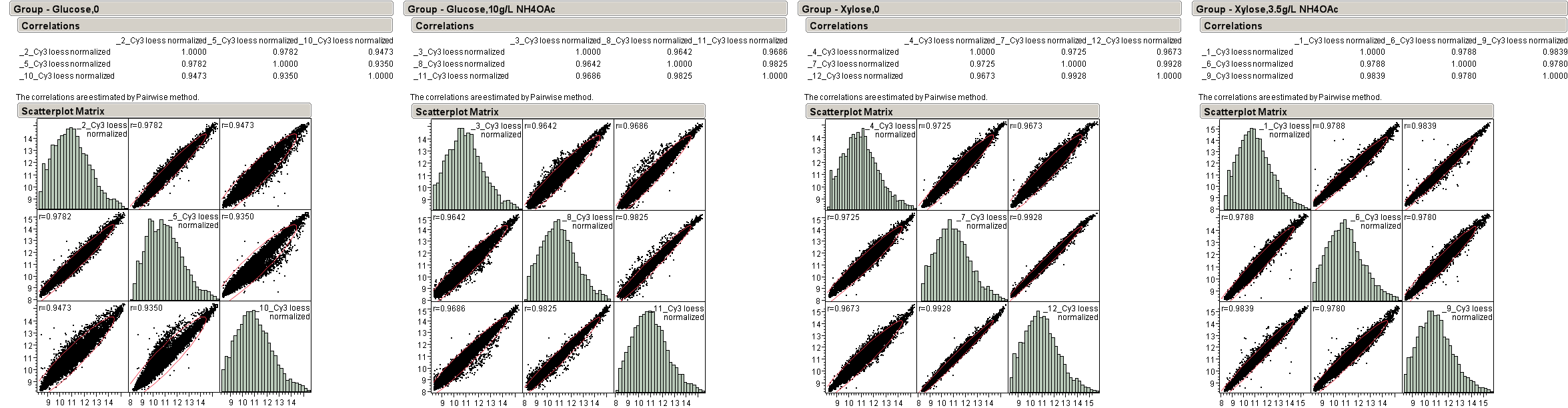


**Glucose Glucose with 10 g/L acetate**

**Xylose Xylose with 3.5 g/L acetate**

**B
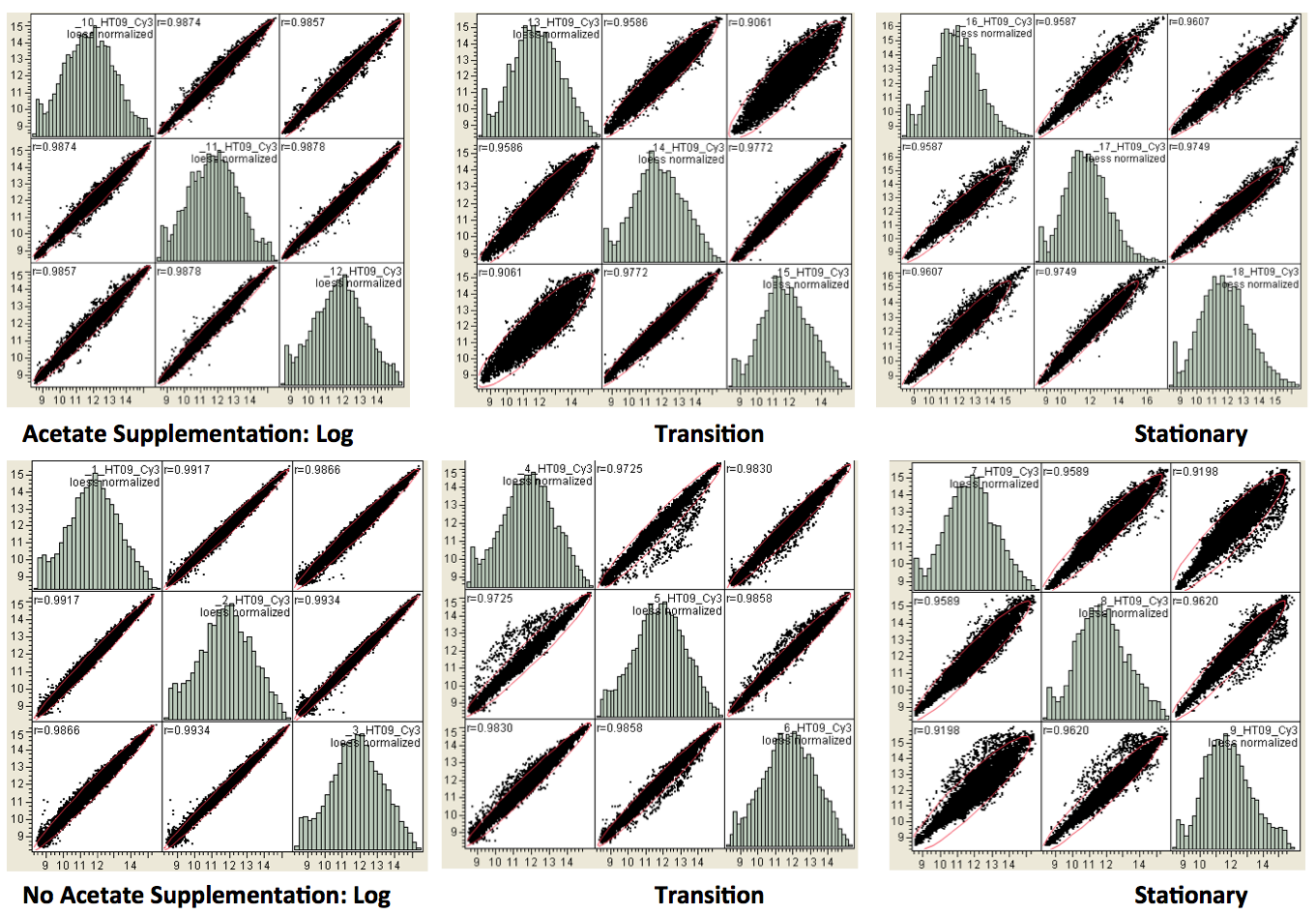
**

**C**
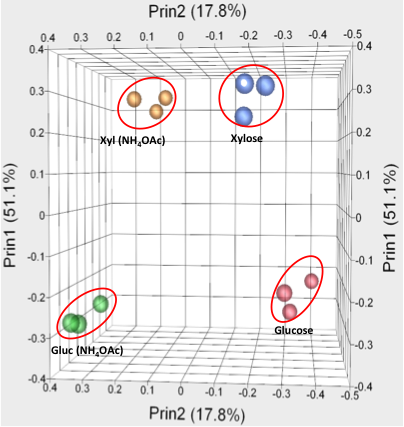
 **D** **
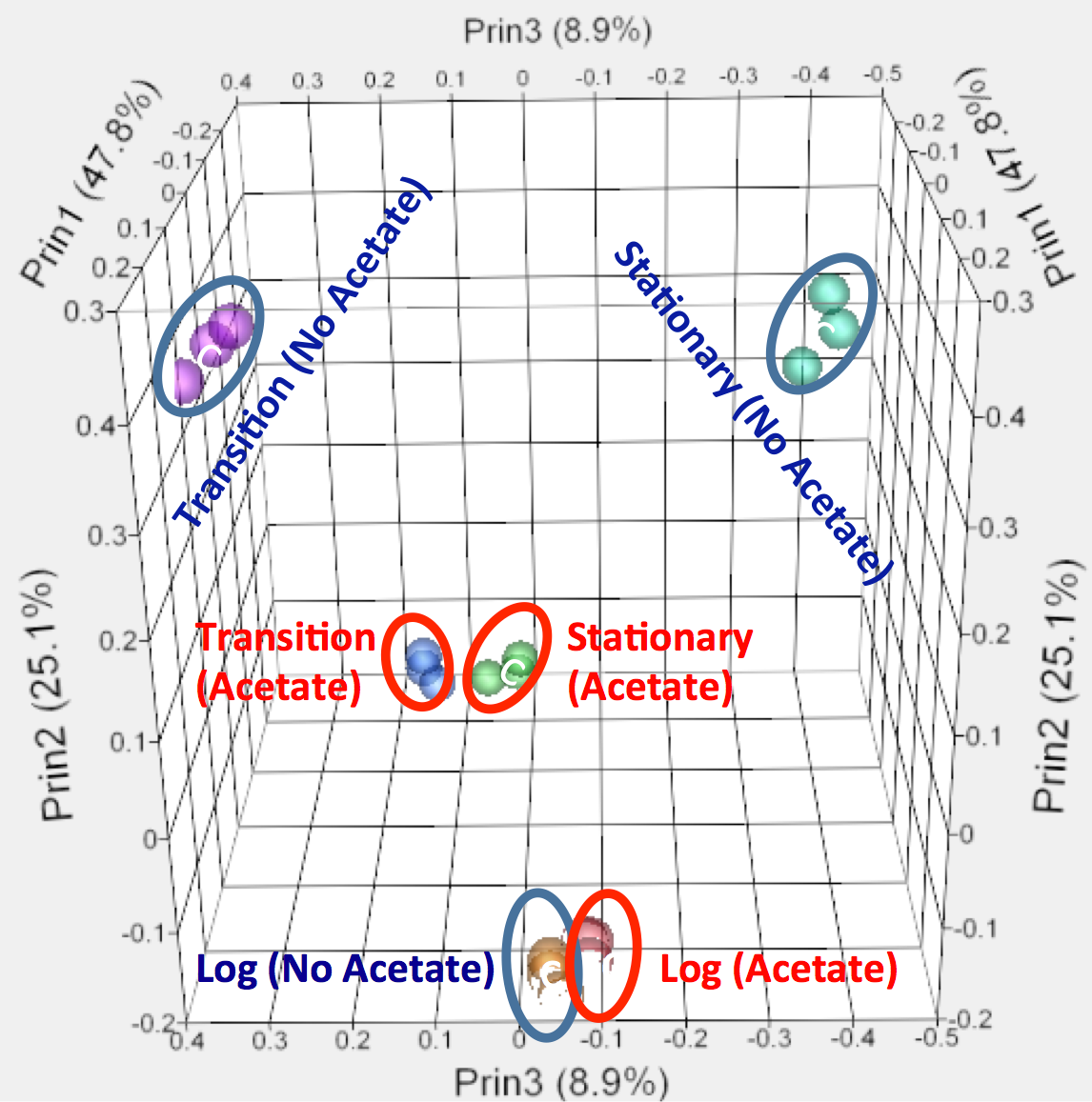
**

**Fig. S1. The quality control analyses of microarray data by JMP Genomics.** Scatterplots showing correlations among biological replicates in the presence and absence of acetate for *Z. mobilis* at exponential phase grown in single sugar media of RMG8 and RMX8 (**A)**, as well as in mixed sugar of RMG4X4 at exponential, transition, and stationary phases (**B)**. The correlation coefficients for microarray data from biological replicates were good, with r > 0.9 for each comparison. In addition, the corresponding principal components analysis results for all samples grown in single sugar of RMG8 and RMX8 (**C)**, as well as in mixed sugar of RMG4X4 at exponential, transition, and stationary phases (**D)** also indicated data had good quality with biological samples from same condition grouped together closely.

**
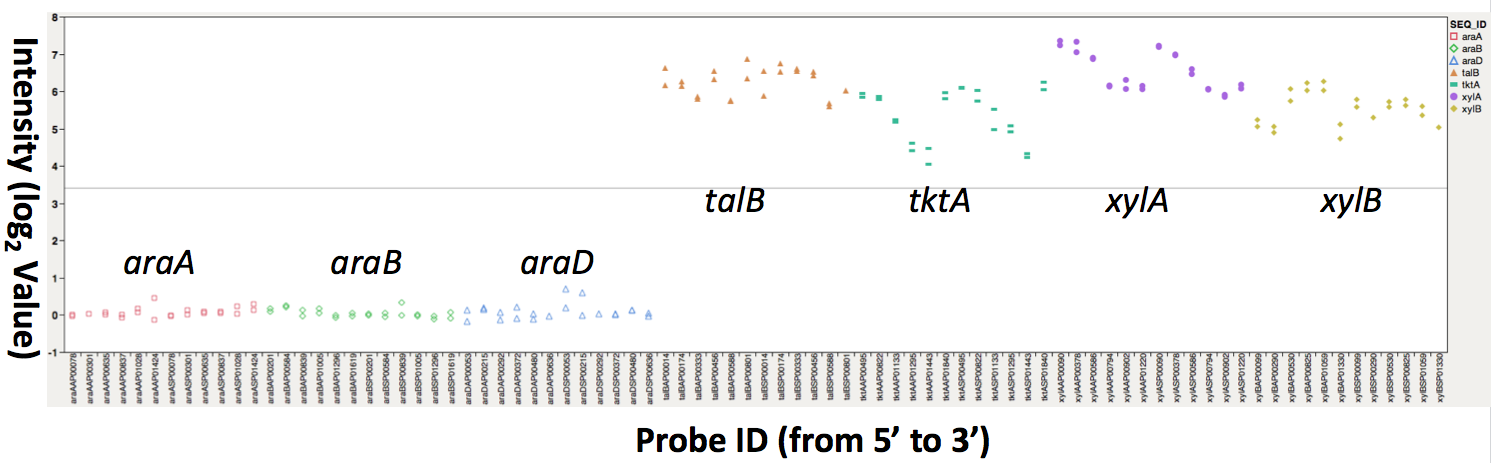
**

**Fig. S2. Example of the recombinant gene expression for *Z. mobilis* in RMG at exponential phase for each probe with two biological replicates list together for each gene.**


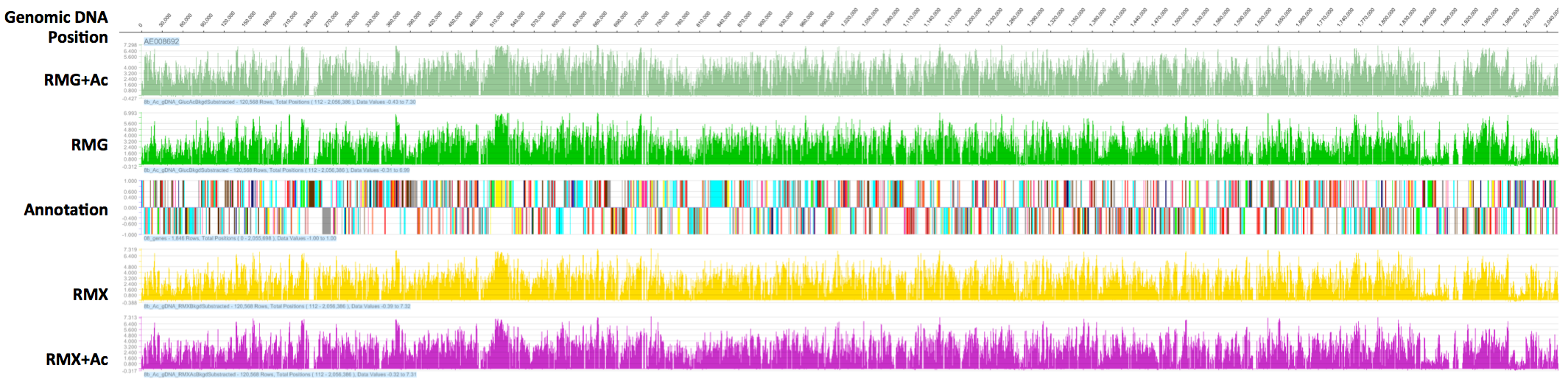


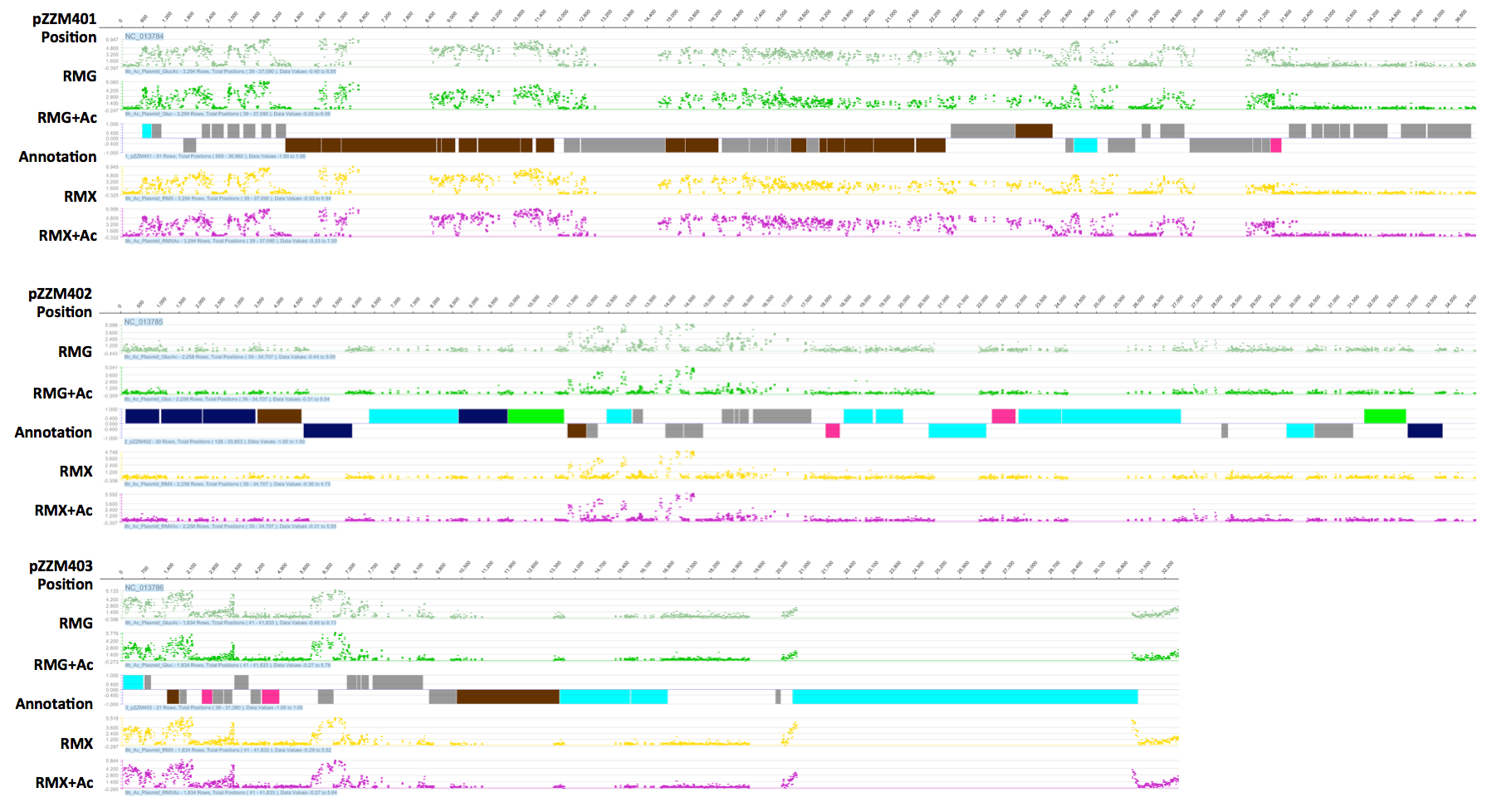

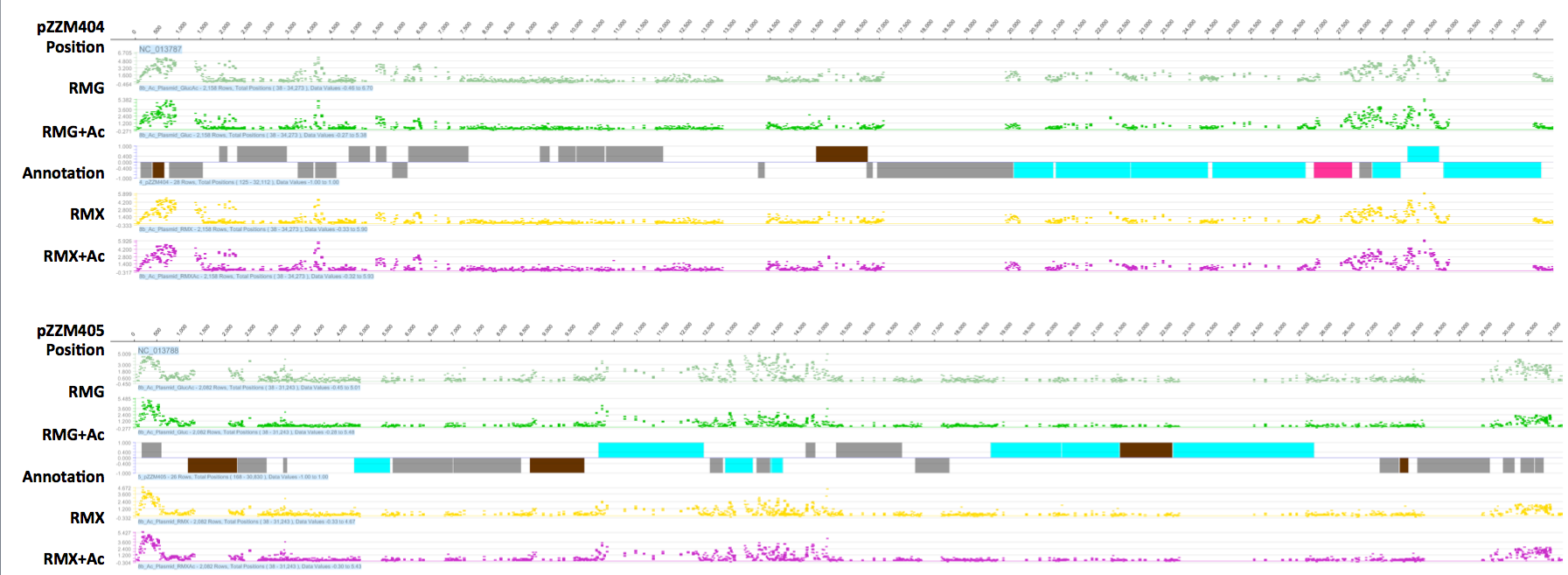


**Fig. S3. Transcriptomic profiles of *Z. mobilis* gDNA and plasmid genes in single sugar of RMG8 or RMX8 with and without the supplementation of exogenous ammonium acetate based on tiling array data.**


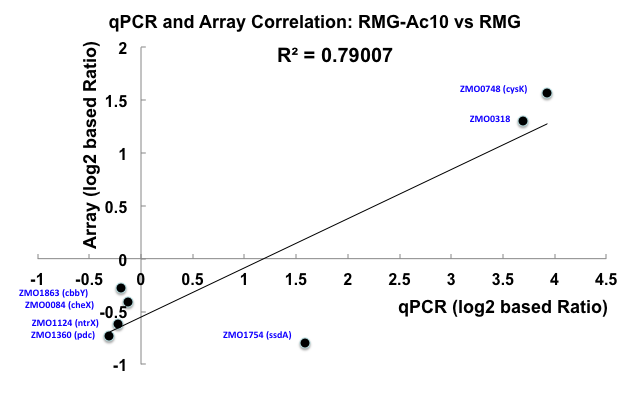
**A**
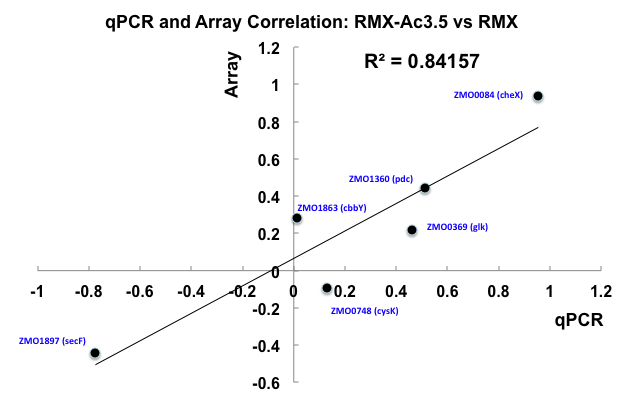
**B**

**
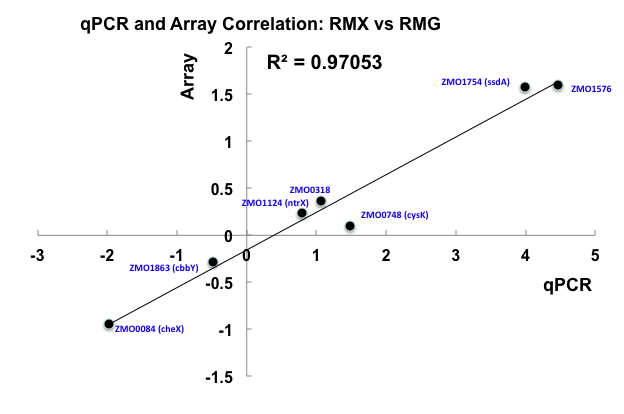
C
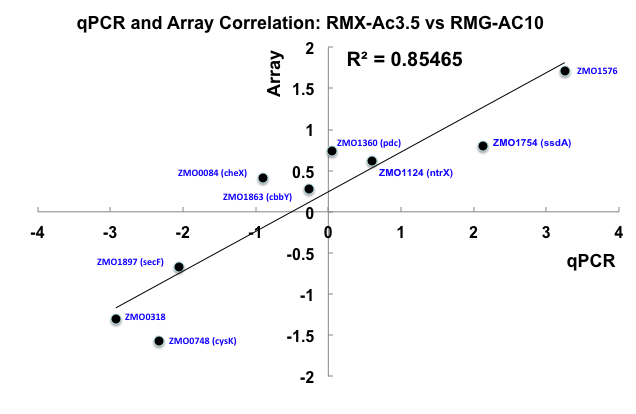
D**

**Fig. S4. The high correlations between qRT-PCR and microarray results indicate good array data. A:** Correlation between qRT-PCR and array data for differentially expressed genes in RMG8 with or without the supplementation of acetate; **B:** Correlation between qRT-PCR and array data for differentially expressed genes in RMX8 with or without the supplementation of acetate; **C:** Correlation between qRT-PCR and array data for differentially expressed genes in RMG8 or RMX8 without the supplementation of acetate; **D:** Correlation between qRT-PCR and array data for differentially expressed genes in RMG8 or RMX8 with the supplementation of acetate.

**A
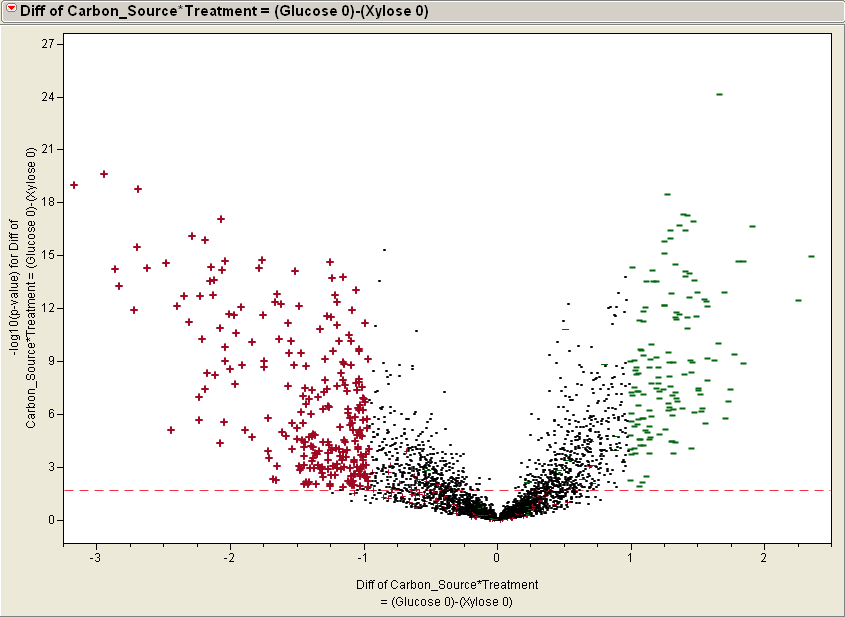
B
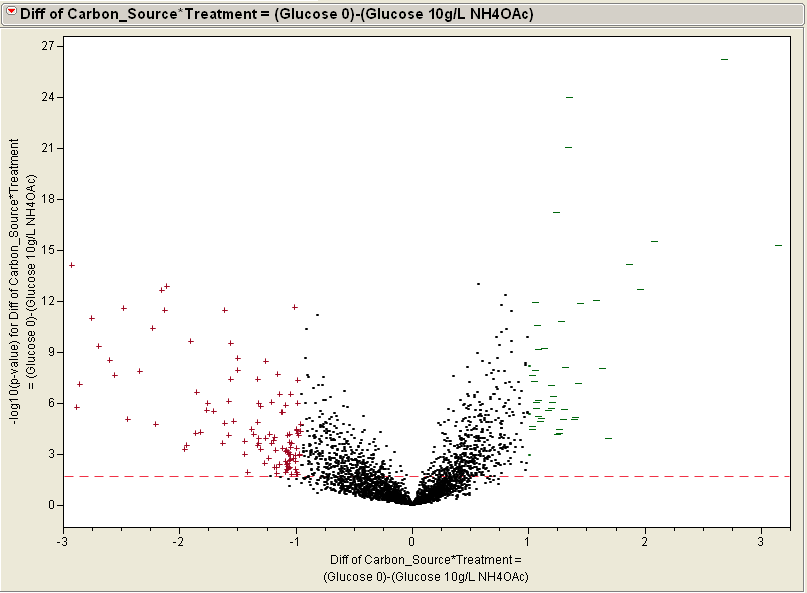
**

**C
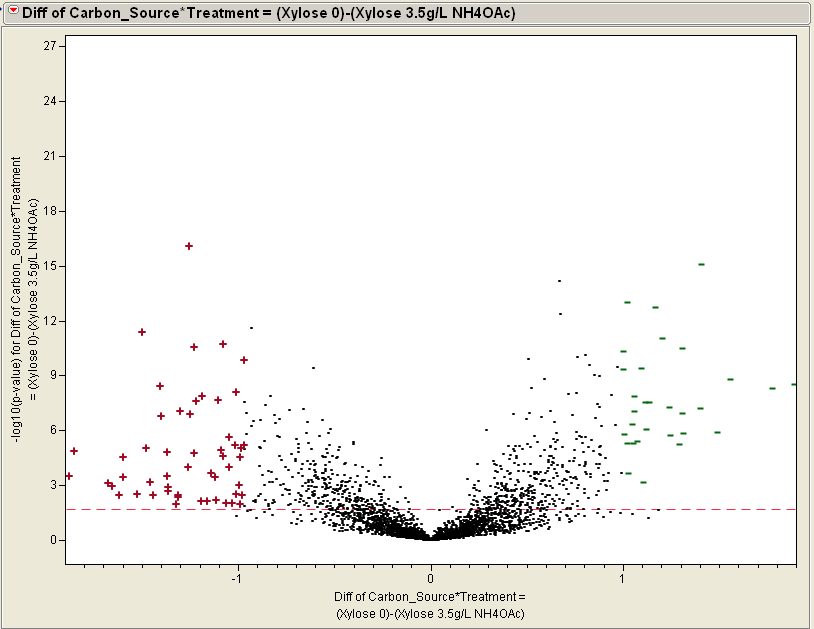
D
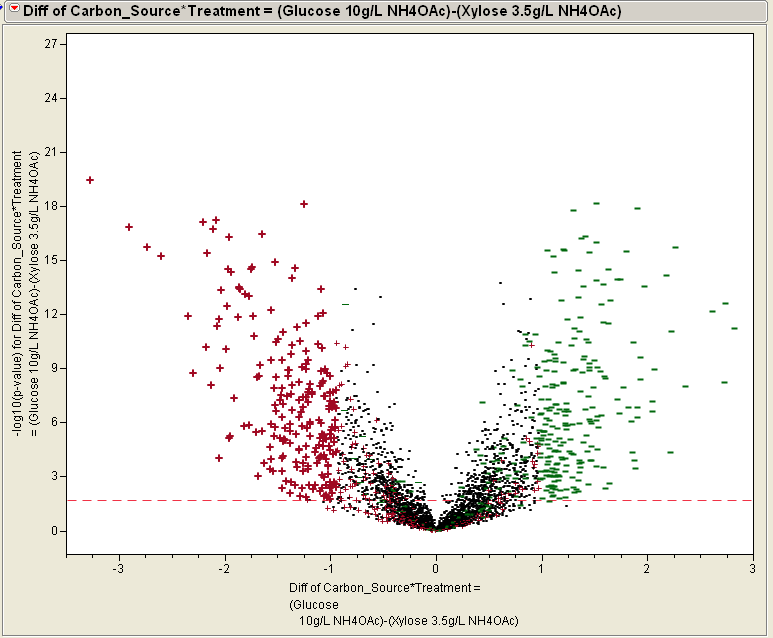
**

**E**
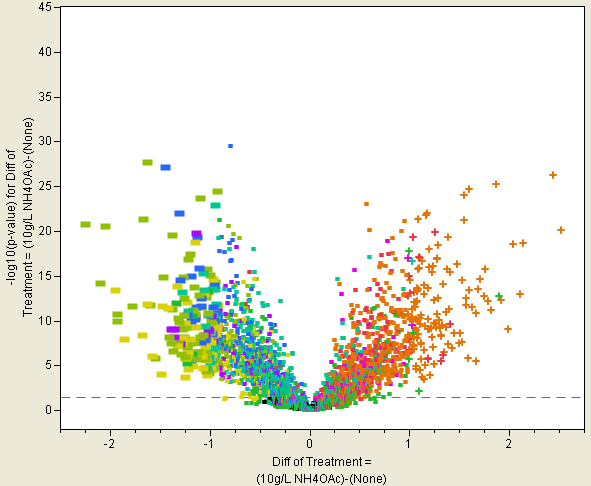
**F
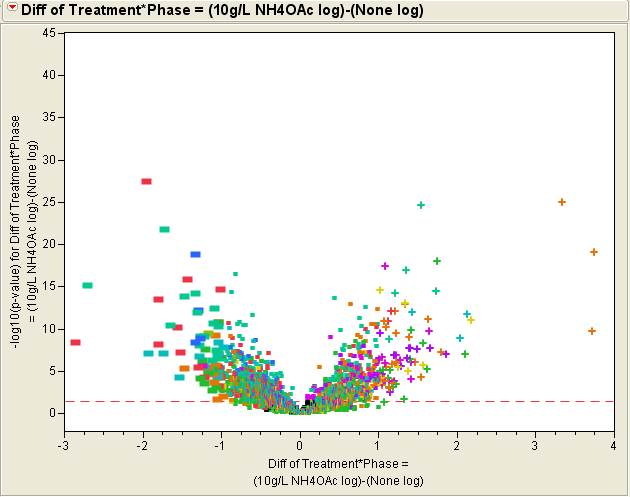
**

**G
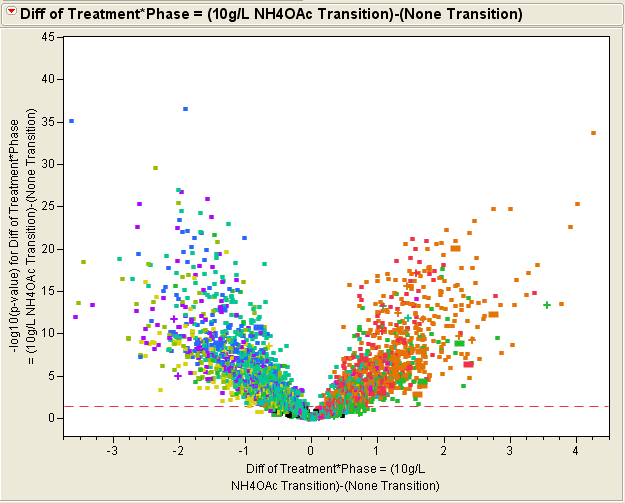
H
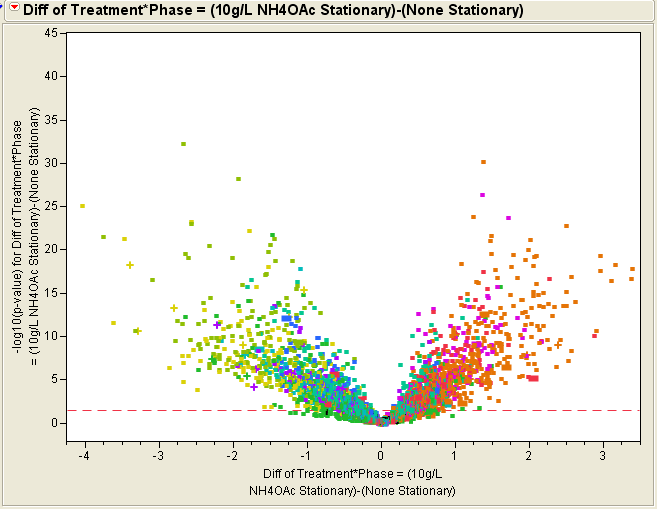
**

**I
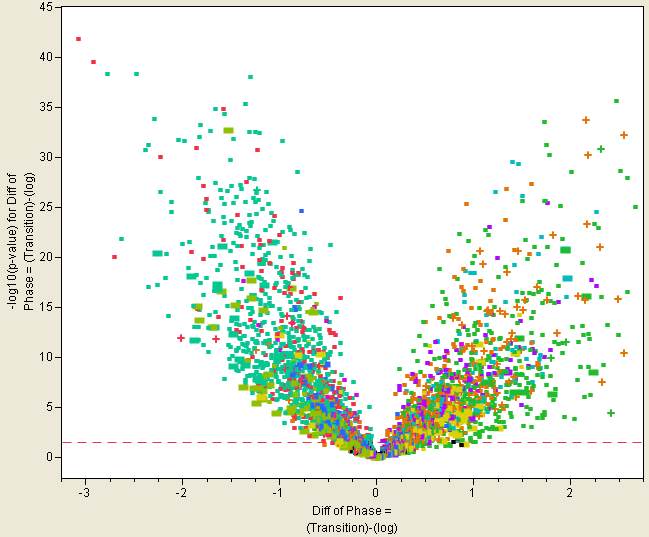
J
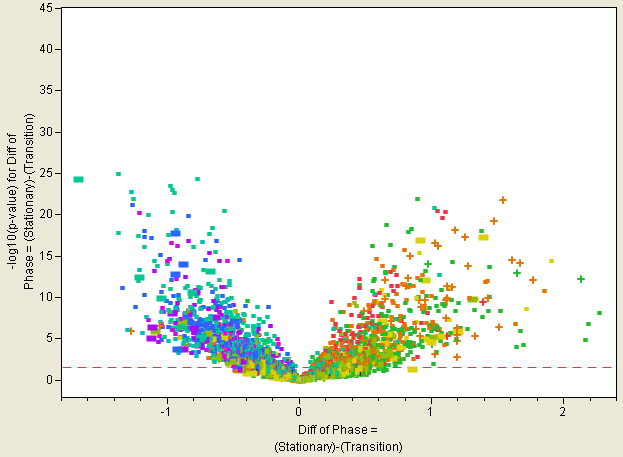
**

**K
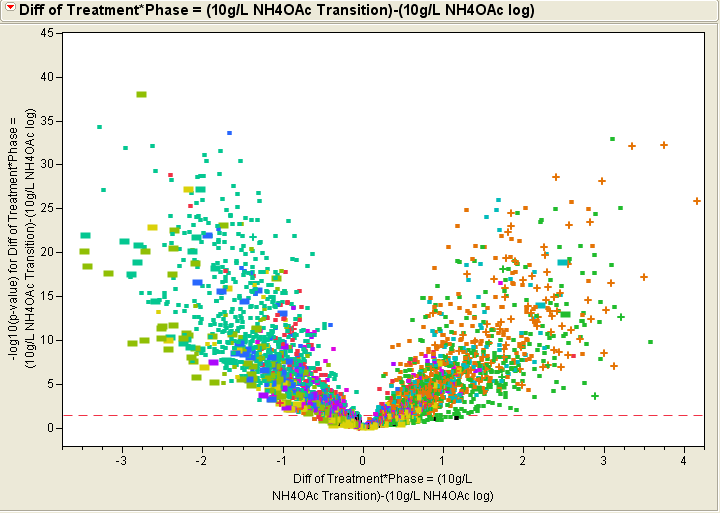
L
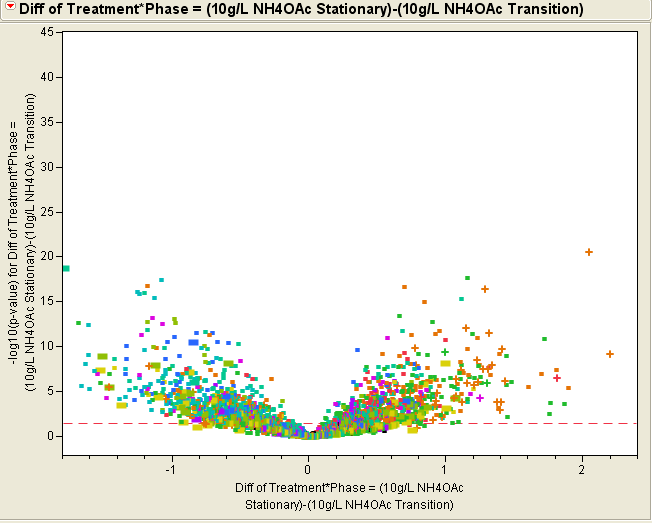
**

**M
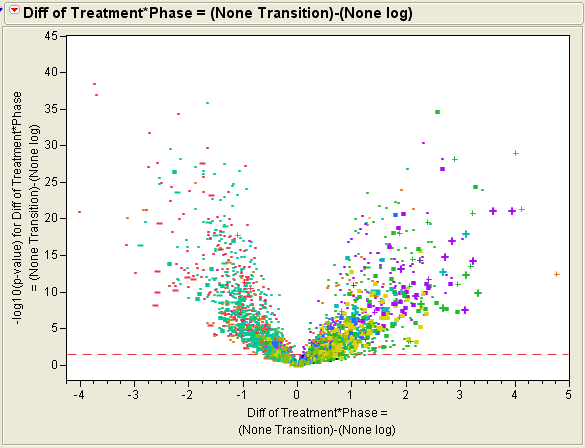
N
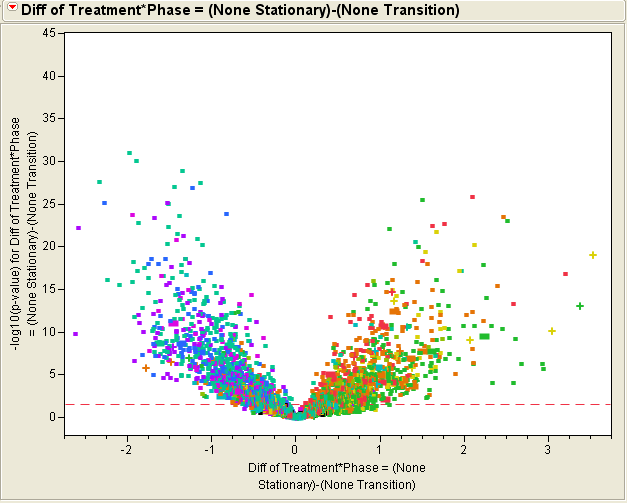
**

**Fig. S5. The ANOVA modeling of acetate shock response microarray data using JMP Genomics** for sugar effect of glucose versus xylose without acetate presence (**A**); Acetate effect in RMG8 (**B**); Acetate effect in RMX8 (**C**); **D:** Sugar effect of Glucose versus Xylose with acetate presence (**D**); Acetate effect in mixed sugar RMG4X4 (**E**), and acetate effect in mixed sugar RMG4X4 in the log phase (**F**), transition phase (**G**), and stationary phase (**H**); as well as time course study of *Z. mobilis* using mixed sugar of RMG4X4 from log phase to transition phase (**I**) and from transition phase to stationary phase (**J**); time course study of *Z. mobilis* using mixed sugar of RMG4X4 with the supplementation of exogenous acetate (10 g/L) from log phase to transition phase (**K**) and from transition phase to stationary phase (**L**); time course study of *Z. mobilis* using mixed sugar of RMG4X4 without the supplementation of exogenous acetate from log phase to transition phase (**M**) and from transition phase to stationary phase (**N**). Each dot represents a genetic feature (either a gene or a intergenic region). Dots above the red dash line are statistically significant features between control and treatment condition. X-axis indicates the log_2_-based ratio between control and treatment condition. Y-axis indicates the statistical significance p-value of –log_10_(P-value).

**A** **
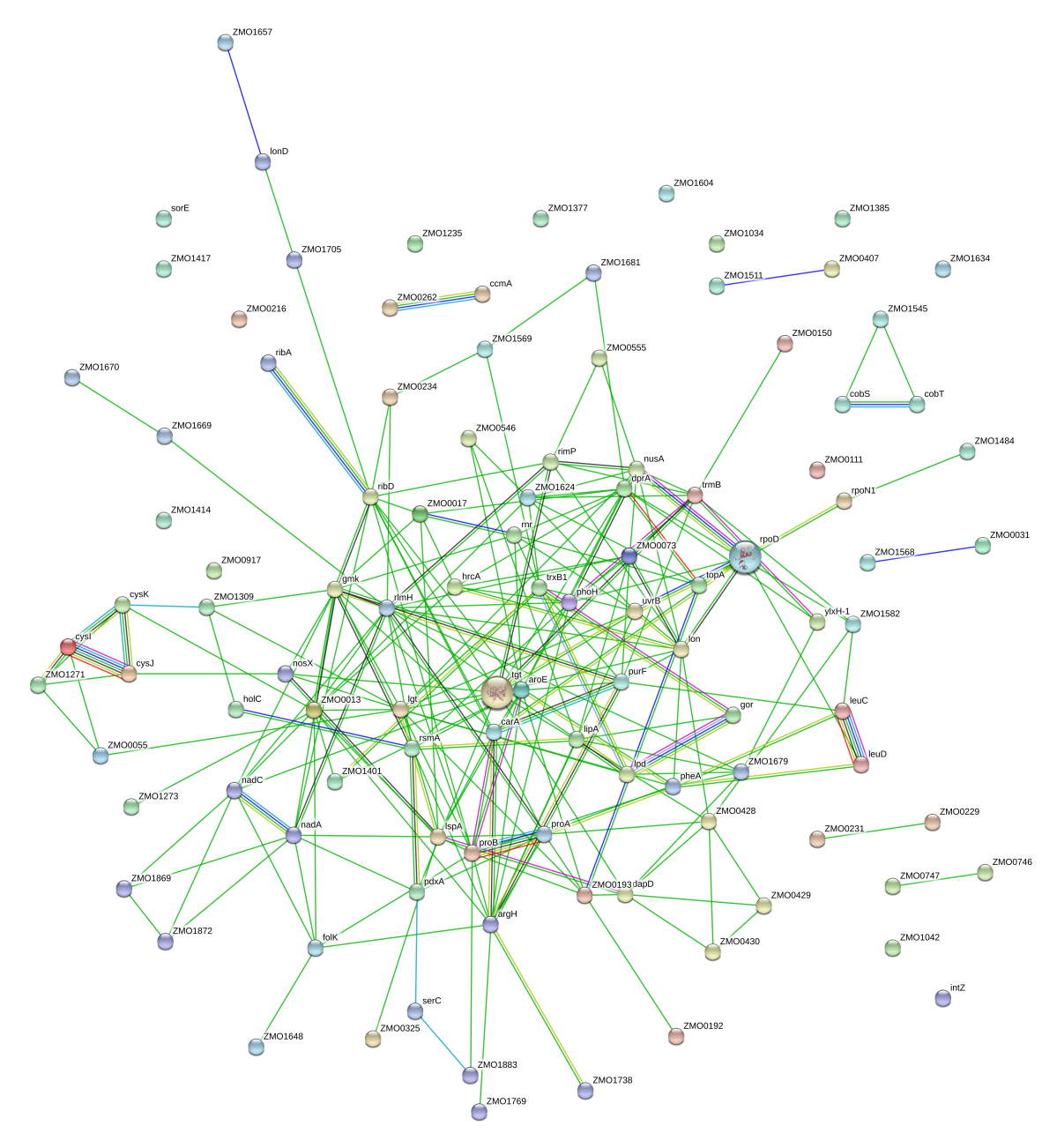
**

**B
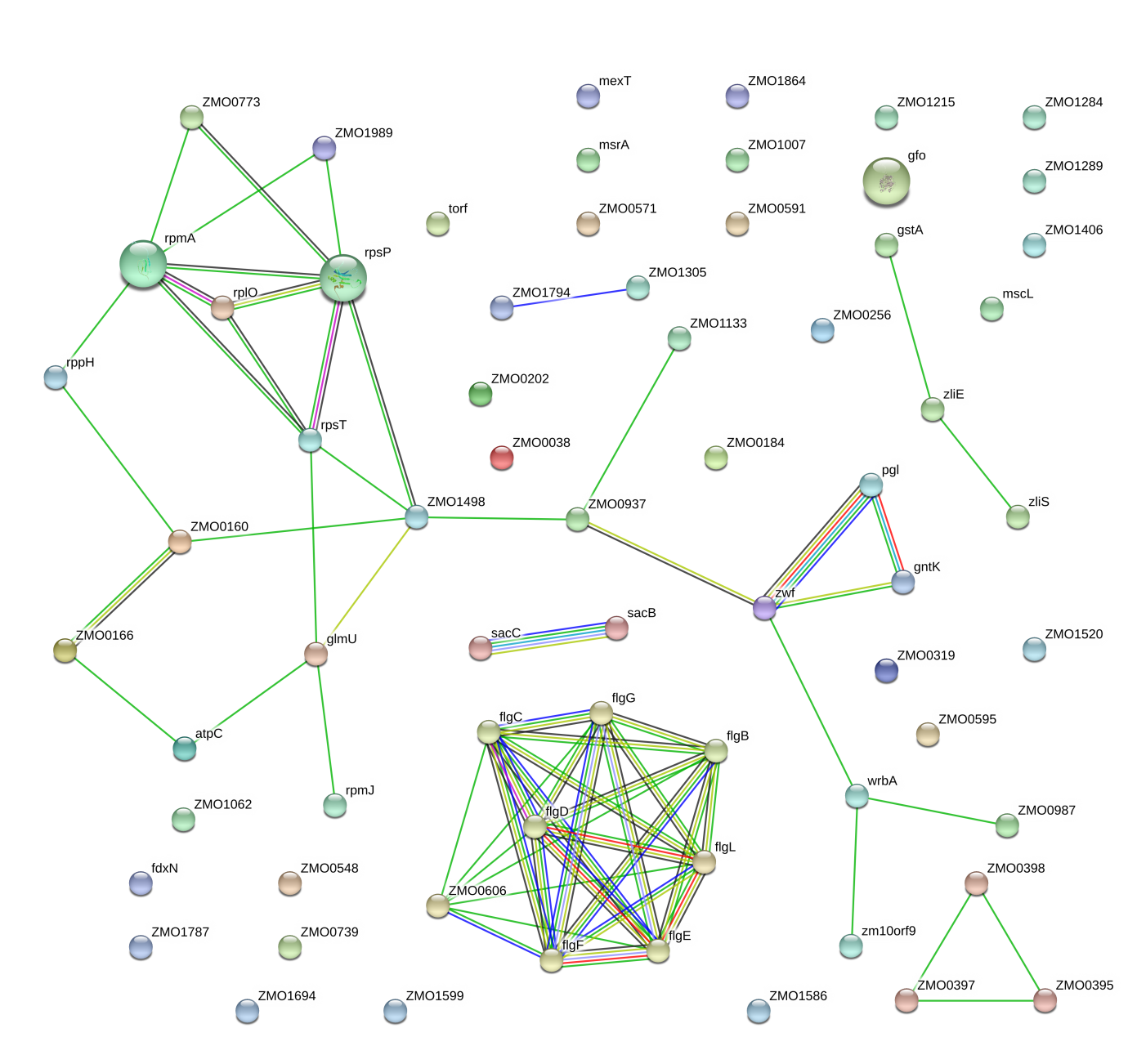
**

**Fig. S6.** Interactions among acetate upregulated genes (**A)** and downregulated genes (**B)** for *Z. mobilis* 8b grown in mixed sugar of RMG4X4 with at least 2-fold significant increase (data from **Table S2-7**) using Strings pre-computed protein-interaction database. Greater numbers of lines are associated with increased connections and greater confident for associations. The network nodes are proteins. The edges represent the predicted functional associations. An edge may be drawn with up to 7 differently colored lines - these lines represent the existence of the seven types of evidence used in predicting the associations. A red line indicates the presence of fusion evidence; a green line - neighborhood evidence; a blue line - coocurrence evidence; a purple line - experimental evidence; a yellow line - textmining evidence; a light blue line - database evidence; a black line - coexpression evidence.


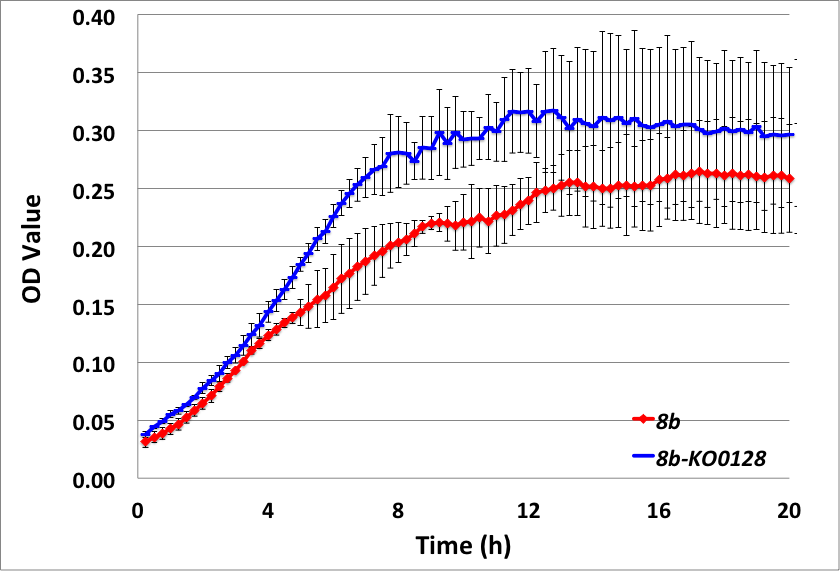


**Fig. S7.** Bioscreen C result of wild-type 8b and ZMO0128 knockout mutant 8b-KO0128 grown in RMG8 with the supplementation of ammonium acetate (15 g/L). At least two independent experiments were carried out with triplicate each time. The paired T-test score between 8b and 8b-KO0128 is 5.3E-82 (*p*≈0) indicating that the difference between 8b and 8b-KO0128 is statistically significant.


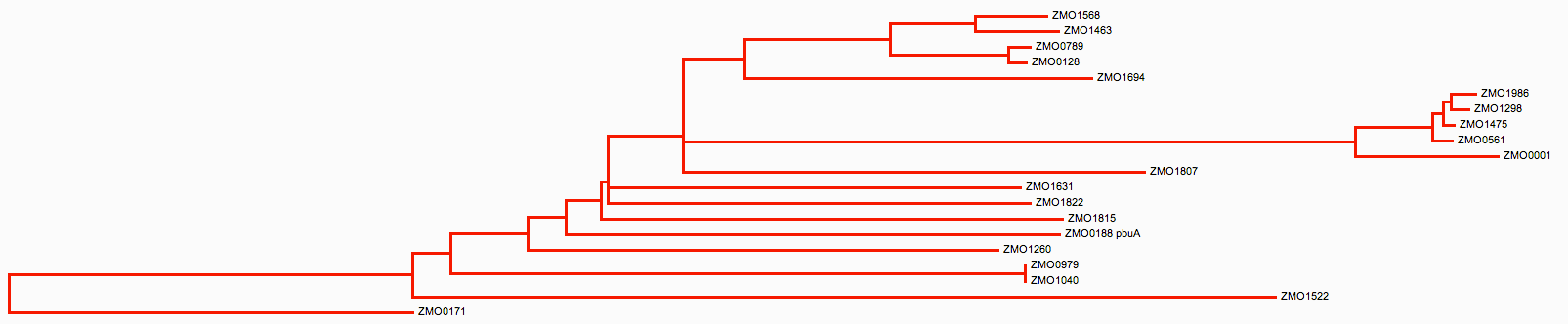


**Fig. S8.** Relationship among TonB-dependent receptors in *Z. mobilis*. Protein sequences were retrieved from *Z. mobilis* ZM4 genome sequence and then aligned using ClustalW program.
