# Supplementary material for: Insights into acetate toxicity in Zymomonas mobilis 8b using different substrates
Source: Biotechnol Biofuels. 2014 Sep 30;7:140. doi: 10.1186/s13068-014-0140-8 (PMC4189746; doi:10.1186/s13068-014-0140-8)
Supplement: Additional file 4: Table S3. — HPLC data of glucose, xylose, xylitol, glycerol, acetic acid, and ethanol (g/L) when 8b grown in RMG8, RMX8, and RMG4X4 media with and without the supplementation of NH4OAc at different time points. [file 13068_2014_140_MOESM4_ESM.docx]

**Table S3.** HPLC data of glucose, xylose, xylitol, glycerol, acetic acid and ethanol (g/L) when 8b grown in RMG8, RMX8, and RMG4X4 media with and without the supplementation of NH_4_OAc at different time points.

| **Description** | **Time**  **(hours)** | **Glucose** | **Xylose** | **Xylitol** | **Glycerol** | **Acetic acid** | **Ethanol** |
| --- | --- | --- | --- | --- | --- | --- | --- |
| **8b (RMG8)** | 0 | 80.48±0.2 | 0±0 | 0±0 | 0±0 | 0±0 | 1.66±0.11 |
|  | 3 | 77.79±0.08 | 0±0 | 0±0 | 0±0 | 0±0 | 3.02±0.23 |
|  | 4.5 | 74.87±0.64 | 0±0 | 0±0 | 0±0 | 0±0 | 4.39±0.34 |
|  | 5.5 | 64.09±1.16 | 0±0 | 0±0 | 0±0 | 0±0 | 9.33±0.65 |
|  | **8** | 0±0 | 0±0 | 0.22±0.3 | 0.34±0.05 | 0.37±0.53 | 38.51±1.05 |
|  | 22 | 0±0 | 0±0 | 0.39±0.01 | 0.33±0.01 | 0±0 | 38.14±0.1 |
|  | 26 | 0±0 | 0±0 | 0.42±0.03 | 0.41±0.09 | 0.34±0.49 | 38.24±0.08 |
| **8b (RMG8-NH4OAc)** |  | **Glucose** | **Xylose** | **Xylitol** | **Glycerol** | **Acetic acid** | **Ethanol** |
|  | 0 | 82.97±0.93 | 0±0 | 0±0 | 0±0 | 0±0.1 | 0±0 |
|  | 3 | 79.65±0 | 0±0 | 0±0 | 0±0 | -0.18±0.03 | 2.45±0.11 |
|  | 4.5 | 77.57±0.16 | 0±0 | 0±0 | 0±0 | -0.28±0.09 | 3.24±0.1 |
|  | 6 | 74.92±0.16 | 0±0 | 0±0 | 0±0 | -0.4±0.09 | 3.86±0.01 |
|  | 8 | 70.31±1 | 0±0 | 0±0 | 0.46±0.06 | -0.33±0.16 | 6.07±0.76 |
|  | 22 | 0.14±0.19 | 0±0 | 0±0 | 0.36±0.04 | -0.48±0.1 | 37.48±0.21 |
|  | 26 | 0±0 | 0±0 | 0±0 | 0.36±0.05 | -0.33±0.17 | 38±0.46 |
|  | **32** | 0±0 | 0±0 | 0±0 | 0.19±0.27 | -0.6±0.48 | 38.24±0.08 |
| **8b (RMX8)** |  | **Glucose** | **Xylose** | **Xylitol** | **Glycerol** | **Acetic acid** | **Ethanol** |
|  | 0 | 0±0 | 76.76±0.44 | 0±0 | 0±0 | 0±0 | 0±0 |
|  | 3 | 0±0 | 74.28±0.4 | 0±0 | 0±0 | 0±0 | 0±0 |
|  | 6 | 0±0 | 73.33±0.38 | 0±0 | 0±0 | 0±0 | 0±0 |
|  | 8 | 0±0 | 73.2±0.28 | 0±0 | 0±0 | 0±0 | 0.67±0.95 |
|  | 22 | 0±0 | 70.45±0.04 | 0.5±0 | 0±0 | 0±0 | 2.73±0.06 |
|  | 26 | 0±0 | 69.57±0.6 | 0.56±0.02 | 0±0 | 0±0 | 3.52±0.32 |
|  | 32 | 0±0 | 67.23±0.08 | 0.6±0.12 | 0±0 | 0±0 | 4.97±0.32 |
|  | 48 | 0±0 | 38±2.16 | 2.38±0.22 | 0.51±0.02 | 1.12±0.07 | 17.56±0.73 |
|  | 72 | 0±0 | 6.9±0.86 | 5.19±0.07 | 0.59±0.16 | 1.01±0.68 | 30.59±0.17 |
|  | 96 | 0±0 | 2.17±0.19 | 5.42±0.04 | 0.68±0.01 | 1.55±0.1 | 33.12±1.09 |
|  | 123.5 | 0.13±0.18 | 1.17±0.03 | 5.39±0.3 | 0.63±0.06 | 1.5±0.41 | 32.5±0.5 |
|  | 148 | 0.22±0.01 | 0.92±0.06 | 5.29±0.21 | 0.54±0.05 | 1.12±0.1 | 32.93±0.31 |
| **8b (RMX8-NH4OAc)** |  | **Glucose** | **Xylose** | **Xylitol** | **Glycerol** | **Acetic acid** | **Ethanol** |
|  | 0 | 0±0 | 75.11±0.08 | 0±0 | 0±0 | 0±0.51 | 0±0 |
|  | 3 | 0±0 | 73.82±0.03 | 0±0 | 0±0 | 0.42±0.02 | 0±0 |
|  | 6 | 0±0 | 71.72±0.75 | 0.21±0.3 | 0±0 | 0.27±0 | 0±0 |
|  | 8 | 0±0 | 72±0.15 | 0±0 | 0±0 | 0.05±0.12 | 0±0 |
|  | 22 | 0±0 | 70.78±0.2 | 0.2±0.28 | 0±0 | 0.18±0.05 | 2.15±0.41 |
|  | 26 | 0±0 | 71±0.34 | 0±0 | 0±0 | -0.16±0.42 | 2.11±0.47 |
|  | 32 | 0±0 | 70.49±0.22 | 0.2±0.28 | 0±0 | 0.09±0.07 | 2.77±0.05 |
|  | 48 | 0±0 | 64.52±1.04 | 0.7±0.01 | 0±0 | 0.2±0.06 | 5.17±0.44 |
|  | 72 | 0±0 | 56.08±1.76 | 0.84±0.04 | 0±0 | -0.22±0.39 | 9.34±1.08 |
|  | 96 | 0±0 | 45.06±3.05 | 1.56±0.01 | 0±0 | -0.1±0.48 | 12.24±1.3 |
|  | 123.5 | 0±0 | 39.9±3.63 | 2.6±0.04 | 0.16±0.23 | 0.07±0.59 | 12.89±1.76 |
|  | **148** | 0±0 | 36.14±3.44 | 3.37±0.03 | 0±0 | -0.31±0.07 | 15.14±1.96 |
| **8b (RMG4X4)** |  | **Glucose** | **Xylose** | **Xylitol** | **Glycerol** | **Acetic acid** | **Ethanol** |
|  | 0 | 37.84±0.52 | 38.12±0.56 | 0.22±0 | 0±0 | 0±0 | 0±0 |
|  | 2 | 38.76±0.27 | 39.7±0.29 | 0±0 | 0±0 | 0±0 | 0.79±0.05 |
|  | 4 | 37.3±0.11 | 39.86±0.25 | 0±0 | 0±0 | 0±0 | 1.76±0.14 |
|  | 6 | 32.02±0.39 | 37.85±0.6 | 0±0 | 0±0 | 0±0 | 4.18±0.67 |
|  | 7 | 28.96±0.81 | 37.75±0.4 | 0±0 | 0±0 | 0±0 | 6.41±0.27 |
|  | 8 | 24.72±0.86 | 37.55±0.04 | 0±0 | 0±0 | 0±0 | 8.99±0.45 |
|  | 10 | 8.35±2.15 | 34.3±0.34 | 0±0 | 0±0 | 0±0 | 17.37±2.83 |
|  | 12 | 0±0 | 29.05±0.83 | 0.15±0.02 | 0.17±0 | 0±0 | 23.52±0.26 |
|  | 21 | 0±0 | 2.59±0.83 | 0.34±0.07 | 0.24±0.02 | 0.47±0.08 | 32.93±0.8 |
|  | 29 | 0±0 | 0.64±0.08 | 0.34±0.08 | 0.3±0.03 | 0.81±0.16 | 34.01±1.58 |
|  | 34 | 0±0 | 0.61±0.24 | 0.36±0.16 | 0.32±0.09 | 0.97±0.25 | 31.9±0.89 |
|  | 36.5 | 0±0 | 0.72±0.18 | 0.34±0.1 | 0.26±0.03 | 0.9±0.15 | 34.13±1.38 |
| **8b (RMG4X4-NH4OAc)** |  | **Glucose** | **Xylose** | **Xylitol** | **Glycerol** | **Acetic acid** | **Ethanol** |
|  | 0 | 37.3±0.75 | 37.55±0.77 | 0.22±0.01 | 0±0 | 0±0.22 | 0±0 |
|  | 2 | 38.19±0.3 | 39.22±0.43 | 0±0 | 0±0 | 0.35±0.08 | 0.78±0.03 |
|  | 4 | 36.91±0.22 | 39.1±0.41 | 0±0 | 0±0 | 0.41±0.13 | 2±0.14 |
|  | 6 | 34.6±0.65 | 38.75±0.48 | 0±0 | 0±0 | 0.4±0.24 | 3.59±0.66 |
|  | 8 | 30.45±2.08 | 37.49±0.32 | 0±0 | 0±0 | 0.16±0 | 5.1±1.09 |
|  | 10 | 25.47±4.3 | 37.12±0.68 | 0.17±0.05 | 0±0 | 0.46±0.04 | 8.9±0.96 |
|  | 21 | 0.25±0.06 | 23.53±2.86 | 0.61±0.1 | 0.16±0 | 0.7±0.13 | 25.84±0.65 |
|  | 29 | 0±0 | 12.35±3.25 | 0.83±0.11 | 0.19±0.04 | 0.79±0.15 | 29.76±1.83 |
|  | 34 | 0±0 | 7.19±2.39 | 0.86±0.1 | 0.18±0 | 0.71±0.35 | 30.41±0.85 |
|  | 36.5 | 0±0 | 3.05±1.26 | 0.84±0.1 | 0.19±0 | 0.87±0.63 | 30.74±1.06 |
|  | 44.5 | 0±0 | 1.82±0.85 | 0.9±0.09 | 0.18±0.01 | 1.52±0.57 | 32.07±2.03 |
|  | 53 | 0±0 | 1.61±0.65 | 0.89±0.13 | 0.18±0.02 | 1.42±0.2 | 31.26±2.19 |
|  | 59 | 0±0 | 1.65±0.67 | 0.86±0.14 | 0.18±0.03 | 1.52±0.22 | 30.66±2.26 |
